# Microbial Quality of Liquid Feed for Pigs and Its Impact on the Porcine Gut Microbiome

**DOI:** 10.3390/ani11102983

**Published:** 2021-10-16

**Authors:** James T. Cullen, Peadar G. Lawlor, Paul Cormican, Gillian E. Gardiner

**Affiliations:** 1 Department of Science, Waterford Institute of Technology, Co. Waterford, X91 K0EK Waterford, Ireland; james.cullen@postgrad.wit.ie; 2 Teagasc, Pig Development Department, Animal and Grassland Research and Innovation Centre, Moorepark, Fermoy, Co. Cork, P61 C996 Cork, Ireland; peadar.lawlor@teagasc.ie; 3 Teagasc, Animal Bioscience Research Centre, Grange, Dunsany, Co. Meath, C15 PW93 Dublin, Ireland; Paul.Cormican@teagasc.ie

**Keywords:** pig, gut microbiome, liquid feed, microbiota, gut health, intestine, microbial quality, fermentation

## Abstract

**Simple Summary:**

Liquid feed is produced by mixing dry feed ingredients with water, and sometimes liquid co-products from the food and beverage industry, at a defined ratio. Liquid feeding of pigs is popular, particularly in parts of northern and western Europe, and can be associated with lower feed costs, improved dry matter intake, growth rate and gut health, compared to dry feeding. However, spontaneous/uncontrolled fermentation upon mixing of feed with water or co-products can decrease the microbial and nutritional quality of the feed, resulting in poorer pig health and growth. For this reason, strategies aimed at optimising liquid feed microbial quality are frequently employed. These include: deliberate fermentation with/without the use of lactic acid bacteria starter cultures that produce lactic acid and lower the feed pH, thereby preventing growth of pathogens. Fermenting only the cereal component of the diet is preferred to whole diet fermentation to minimise loss of free amino acids from the diet during fermentation. This review examines the microbiome of liquid feed and explores how optimisation strategies impact both feed microbial quality and the gut microbiota and growth of liquid-fed pigs. It also covers cleaning and disinfection of liquid feeding systems and how this might impact liquid feed microbial quality.

**Abstract:**

There is evidence that spontaneous fermentation frequently occurs in liquid pig feed that is intended to be delivered as fresh liquid feed, often with a resultant deterioration in the microbial and nutritional quality of the feed, which can negatively affect pig health and growth. Strategies including controlled fermentation with microbial inoculants, pre-fermentation or soaking of the cereal fraction of the diet, enzyme supplementation and dietary acidification have been employed to inhibit pathogens and prevent deterioration of feed nutritional quality, with promising results obtained in many cases. This review evaluates the impact of these strategies on the microbial quality of liquid feed and discusses how they can be further improved. It also investigates if/how these strategies impact the pig gut microbiota and growth performance of liquid-fed pigs. Finally, we review liquid feed system sanitisation practices, which are highly variable from farm to farm and discuss the impact of these practices and whether they are beneficial or detrimental to liquid feed microbial quality. Overall, we provide a comprehensive review of the current state of knowledge on liquid feed for pigs, focusing on factors affecting microbial quality and strategies for its optimisation, as well as its impact on the pig gut microbiome.

## 1. Introduction to Liquid Feed

In this review, liquid (or wet) feed refers to a mixture of dry feed components (cereals, protein sources and pre-mixes containing vitamins, minerals, synthetic amino acids and other feed additives) combined with either water and/or liquid food industry co-products (e.g., dairy and distillery co-products), in a mixing tank to a pre-defined water:feed ratio, prior to feed-out. The homogenised liquid feed is pumped from the mixing tank to troughs located in pig pens via a network of pipes [1,2]. Such feeding systems are computer-controlled and frequently referred to as automated liquid feeding systems.

**Figure 1:**
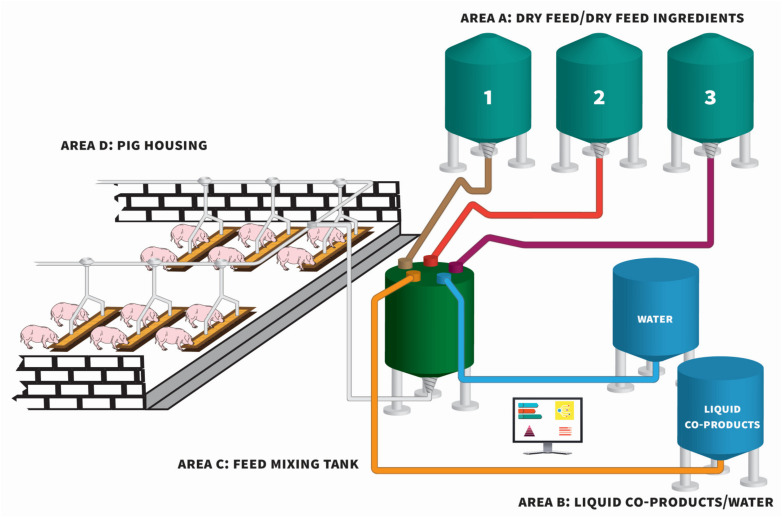
Diagram of an automated liquid feeding system demonstrating how dry feed/dry feed ingredients from feed bins (Area A) and water and/or liquid co-products (Area B) are delivered to a central mixing tank (Area C) and agitated, followed by the delivery of liquid feed to pens via a series of pipes for consumption by pigs (Area D). On farms where fermentation is practiced, an additional fermentation tank may be included before the mixing tank, where the whole diet or the cereal fraction of the diet are fermented for a period of time prior to pumping to the mixing tank for delivery to the pens.

Liquid feeding is generally carried out using either long or short troughs. The former allows all pigs in the pen to eat simultaneously and because of this, the feed allowance to pigs can be restricted. However, with the latter, only a portion of the pigs in a pen (normally 30–40%) can eat at any one time and so pigs must be provided with *ad libitum* access to feed which is controlled by readings from sensor(s) or probe(s) within the trough to maintain a reservoir of feed within the trough [3]. Figure 1 illustrates a typical modern liquid feeding system. The distinction between liquid feeding and wet/dry feeding should be noted; with wet/dry feeding, dry feed and water remain separate before entering the trough, where the pig can then mix them at its desired water:feed ratio [4]. 

### 1.1. Prevalence of Liquid Feeding

Liquid feeding is common in many parts of the world, most notably in western Europe [3]. However, there is a lack of up-to-date accurate information regarding the number of liquid-fed pigs in Europe. Approximate figures from Best [5] indicate that >60% of Danish and Swedish finishers, as well as the majority of sows, are liquid-fed. Best [5] also reported that approximately one third of grow-finisher pigs in the Netherlands and France are liquid-fed, but in the main pig regions this figure is between 50–60%; however, Martineau et al. [6] reported that ~70% of finishers in France are liquid-fed. About 40% of grow-finishers in Germany receive liquid diets but the figure is much lower for sows [5]. Data collected from a survey of 56 Irish farrow-to-finish pig farms found that 37.5% of these fed a liquid diet from weaning to slaughter [7]. However, it should be noted that in Ireland it is on the farms with large herd sizes that liquid feeding is most prevalent. Therefore, in order to obtain a truer picture of the prevalence of liquid feeding, rather than basing it on the number of herds/farms using the practice, it should be calculated based on the number of pigs that are liquid-fed. In Ireland this figure is estimated to be ~70% [8]. Liquid feeding is less popular in North America, compared to Europe. However, an exception to this is Ontario, Canada, where in 2012, 20% of grow-finishers were fed liquid diets, but with the difference being that these are primarily corn-based, compared to the mainly wheat- and barley-based diets fed in Europe [3]. Liquid feeding has been adopted more readily in Europe due to the widespread availability of inexpensive nutrient-rich co-products from the food, beverage and biofuel industries, which aid in reducing feed costs by up to 17% compared to dry feed [3,9,10]. These will be discussed in more detail in Section 2.2.

### 1.2. Types of Liquid Feed

The two main types of liquid feed (LF) are fresh LF and fermented LF (FLF). Liquid feed is characterised as fresh when the whole diet is mixed with water/liquid co-products, usually at a ratio of 1:1.5 to 1:4, immediately prior to feeding. It has been well documented, however, that some degree of unintentional ‘spontaneous’ fermentation occurs in fresh LF once mixing begins. This may have a negative impact on the microbial quality of the feed as a result of malfermentation. This occurs due to the selection for, and proliferation of, undesirable microbes and subsequent microbial degradation of crystalline amino acids in the diet, leading to the production of undesirable metabolites such as biogenic amines [2,11,12,13]. On the other hand, FLF is deliberately fermented, either with/without the use of a microbial inoculant. Water/liquid co-products are mixed with the whole diet (or the cereal fraction alone), at a similar ratio to fresh LF, and the mixture is allowed to ferment for a period of time prior to feeding, with the addition of the remaining dietary components just prior to feed-out in the case of fermentation of the cereal fraction [13,14].

A common method of producing FLF is the mixing of fresh feed and liquid with a proportion of a previously successful fermentation; a process known as ‘backslopping’. The production of organic acids, such as lactic acid, produced by lactic acid bacteria (LAB) fermentation is considered one of the key benefits of FLF, as it reduces the pH of the feed and the pig gastrointestinal (GI) tract, resulting in a reduction in the levels of *Enterobacteriaceae* [15,16]. An alternative to microbial fermentation of LF is the direct addition of organic acids to fresh LF to produce acidified LF (ALF). The potential benefits and disadvantages of the aforementioned LF types will be discussed in detail throughout this review, along with their microbial quality, and their impact on the pig gut microbiome and pig growth.

### 1.3. Desirable Characteristics of Liquid Feed

The widely accepted desirable characteristics of FLF include low pH (generally <4.5), high numbers of LAB, low numbers of *Enterobacteriaceae*, high concentrations of lactic acid and low concentrations of acetic acid [17,18,19]. Fungal communities are also an important component of LF, with positive or negative impacts on feed quality, depending on the species dominating [20]; however, excessive yeast fermentation is generally undesirable as high levels can impact feed palatability due to the production of acetic acid and ethanol in addition to a loss of energy from the feed [2,14]. Olstorpe et al. [21] suggested adding a desirable yeast strain to LF starter cultures that could dominate the feed without reducing feed microbial and nutritional quality. Certain fungal species have potential benefits in LF including acting as a protein source [22,23] and inhibiting moulds and *Enterobacteriaceae* [24,25]. The potential benefits of fungi needs to be further explored as there is evidence to suggest that yeast species such as *Kazachstania slooffiae* are beneficial to pig gut health, providing amino acids for microbial as well as pig growth and exhibiting a potential symbiotic relationship with *Lactobacillus* [26,27,28].

Van Winsen et al. [15] described a successful batch of FLF as having: pH <4.5; lactic acid >150 mmol/L; acetic acid <40 mmol/L; butyric acid <5 mmol/L; ethanol <0.8 mmol/L; total lactobacilli >9 log_10_ CFU/mL; *Lactobacillus plantarum* >9 log_10_ CFU/mL; *Enterobacteriaceae* <1.8 log_10_ CFU/mL; and no detectable *Salmonella*/25 mL. Standard microbiological and physicochemical properties of ‘residue-free’ and ‘residue-containing’ LF can be seen in Table 1. The former refers to a situation where the pipelines are rinsed with water prior to delivery of the new batch and is akin to fresh LF. The latter refers to a situation where residual feed from a previous batch remains in the pipelines and is recirculated with the new batch of feed, thus acting as an inoculum for fermentation (in a similar fashion to backslopping). Therefore, it is essentially FLF, as evidenced by its properties (Table 1). However, in practice, farms operating both of these systems will likely consider that they are both feeding fresh LF as neither intentionally sets out to produce FLF.

## 2. Potential Benefits of Liquid Feed for Pigs

Liquid feeding has several potential advantages compared to conventional dry feeding of pigs. These include improved gut health, utilisation of inexpensive industry co-products, flexibility and ease of delivery, and the ability to optimise microbial and nutritional quality via the addition of feed additives such as microbial inoculants for controlled fermentation and enzyme preparations to improve nutrient digestibility [3,31,32,33]. These advantages can improve the growth and feed efficiency (FE) of pigs, while different liquid feeding strategies may also act as suitable alternatives to the traditional inclusion of sub-therapeutic levels of in-feed antibiotics and pharmacological levels of zinc oxide [2,11,20].

### 2.1. Improved Gut Health and Pathogen Inhibition

As outlined earlier, the mixing of feed and water during LF preparation allows for the proliferation of naturally occurring fermentative LAB and yeast present in feed ingredients. The phase of fermentation at which FLF is fed is important, as during the initial phase, conditions are conducive to a rapid surge in coliforms and other potential pathogens [10]. Optimal conditions are achieved once the fermentation reaches ‘steady state’ at the end of the second phase and into the third phase of fermentation, when there are high levels of LAB and lactic acid, moderate levels of yeast, low pH, and low numbers of enterobacteria [11,14,31]. At this stage, the production of lactic acid, acetic acid and ethanol by the dominant microbiota, has reduced the pH, preventing pathogens such as *Escherichia coli* and *Salmonella* from proliferating [20]. During the third phase, however, yeasts can continue to proliferate in the feed [14]. There is also evidence that when fed to pigs, low pH liquid feed can increase lactic acid concentrations in the stomach, reduce gastric pH, and reduce enterobacteria along the GI tract [11,34]. The effect of liquid feeding on the pig gut microbiota will be discussed in Section 6. The health benefits for liquid-fed pigs have been reviewed by Brooks et al. [14,35] and include reduced *Salmonella* prevalence, reduced diarrhoea incidence and a reduction in antibiotic-resistant *E. coli.*

### 2.2. Use of Industry Co-Products

The inclusion of inexpensive food and beverage industry co-products in animal diets has traditionally been used as a means of decreasing feed cost and as an alternative to disposal, which has an associated economic, as well as environmental impact [9,36]. The use of industry co-products, however, requires careful management of diet formulation as co-products such as whey can be high in salt, and may also increase water requirements of pigs [3]. Another challenge of co-product inclusion is the variability in microbial and nutritional composition between different products and indeed batches of the same co-product [37]. Nonetheless, depending on availability, continuity and consistency in supply and proximity of co-products to a given farm, it can be a viable means of reducing feed costs. Another potential benefit of liquid co-product inclusion is that many sugar-rich food and beverage industry co-products have undergone fermentation by LAB and/or yeast and, therefore, have a pH of ~3.5–4.5 resulting from the formation of organic acids. The resultant lactic acid, and to a lesser extent acetic acid, are known to exhibit antimicrobial activity against pathogenic and spoilage microorganisms [9,14]. In a survey of liquid feeding practices in the finisher section of commercial Irish pig units, O’Meara et al. [12] found that 3 of the 8 units surveyed included either pot-ale syrup and/or liquid whey in their diets. The study found that on these pig units, *Enterobacteriaceae* counts in LF delivered freshly to troughs tended to be lower compared to units that did not use co-products. Additionally, *E. coli* counts were reduced in residual LF remaining in troughs just prior to the next feed-out on the units that used liquid co-products. Dietary co-product inclusion also reduced mould counts in the mixing tanks, as well as in fresh and residual LF in troughs, while also reducing the pH of the feed in the mixing tanks and of the fresh feed in the troughs [12].

### 2.3. Other Benefits of Liquid Feed

#### 2.3.1. Reduced Feed Costs

Recently, Lawlor [38] examined the effect of different feeding systems on margin over feed per pig during the growing finishing stage. This analysis was based on finisher feed price and pig meat price in Ireland in August 2021 (Teagasc feed and pig-meat price monitor, 2021) and data from O’Meara et al. [39]. It was found that in order to reach a target slaughter weight of 105 kg, where finisher space is not limited, dry and wet/dry feeding resulted in a higher margin over feed than liquid feeding because of superior FE in the former. However, where farms are limited by space and, therefore, maximising growth rate is essential to reach a target slaughter weight, as is the case on many finisher units, then liquid feeding is as cost-effective as dry and wet/dry feeding, due to the increased growth rate observed with liquid feeding. These results are in agreement with a similar analysis performed in 2018 [8]. The real benefit of liquid feeding over dry and wet/dry feeding, however, is that it allows the inclusion of low to medium dry matter (DM) co-products in the diet which can greatly reduce feed cost.

#### 2.3.2. Practical Benefits

In addition to the improved DM intake and growth rates observed with liquid feeding [20,35,40], there are a number of practical benefits over dry feeding which include the ability to optimise microbial and nutritional quality via addition of feed additives such as starter cultures for controlled fermentation, enzyme preparations to improve nutrient digestibility, and direct acidification of feed using organic acids [16,41]. Liquid feeding systems also allow for increased accuracy of feeding, as more appropriate feeding curves can be achieved [35] while different diets can also be fed to different pens allowing for phase feeding [13,40]. If home compounding, ingredients can be mixed to form a diet prior to feeding, and thus the liquid feeding system acts as both a feed mixing and distribution system [40]. Liquid feeding also reduces dust during handling and feeding compared to dry feeding, resulting in less feed loss and a healthier environment for the stockperson and the pigs [13,35].

## 3. Disadvantages of Liquid Feed for Pigs

### 3.1. Formation of Biogenic Amines/Loss of Nutritional Value

Concerns over biogenic amines in LF include decreased nutritional value of feed due to microbial decarboxylation of free amino acids as well as toxicity and reduced feed palatability with a subsequent reduction in feed intake [2,42,43]. However, the only relevant EU legislation is concerned with histamine content of food, and no clear guidance exists on acceptable levels of biogenic amines in LF [44]. A recent cytotoxicity study by del Rio et al. [45] found that the highest levels of putrescine and cadaverine that elicited no adverse effects on an intestinal cell line were 440.75 and 255.45 mg/kg body weight/day, respectively. A French survey of 33 finishing units reported 310 and 1182 ppm (mg/kg) as the maximum detected levels of putrescine and cadaverine in LF for pigs, respectively, indicating that amine levels in LF, particularly cadaverine, may be a concern. They also suggested that biogenic amine levels in LF are highly variable and linked to individual farms, with the use of industry co-products, for example, being a risk factor for high levels [46].

The microbial decarboxylation of free lysine, which is usually added to pig diets as lysine-hydrochloride (lysine HCl) to fulfil nutritional requirements, results in the formation of cadaverine, with some cadaverine formation also occurring due to ornithine decarboxylation [47]. Putrescine is also formed from the decarboxylation of ornithine and/or arginine [48]. The amino acid decarboxylase enzymes required for biogenic amine formation are widely distributed among different bacterial groups including undesirable and spoilage-associated microbes such as members of the *Enterobacteriaceae* family. However, they are also produced by many desirable microorganisms such as LAB which are naturally present in feed, or may be intentionally added as an inoculum; however, this characteristic is strain-specific [44,48,49]. This highlights the need for careful consideration when choosing a microbial inoculum for FLF.

### 3.2. Other Disadvantages of Liquid Feed

Despite the higher DM intake of pigs fed LF compared to dry feed, feed conversion efficiency (FCE) is usually poorer, primarily due to feed wastage, especially with short trough/*ad libitum* liquid feeding [50,51]. Issues with feed palatability and thus feed refusals can also occur with LF, usually due to the formation of acetic acid and ethanol resulting from yeast blooms [2,40]. The DM content of LF is limited by the capacity of the liquid feeding system to pump the mixture through the pipelines [1]. Older liquid feeding systems with less efficient pumps and more bends in the piping may require the use of excessive water:feed ratios to reduce the viscosity of LF mixtures. This results in reduced growth and poorer FE due to reduced feed intake (which is limited by the intake capacity of the pigs) as well as energy being expended to excrete excess water [1]. Additionally, this results in increased manure production and therefore higher manure transport and storage costs due to a more dilute nutrient content [2,50]. The use of liquid co-products also requires on-farm storage, which incurs an extra cost. There is also the risk of spoilage during storage, with the potential for deterioration of nutritional quality and energy loss, thereby requiring a high level of quality control and management when formulating diets [3,52].

## 4. Microbial Quality of Feed

### 4.1. Dry Feed; Meal Versus Pellets

Liquid feed is generally prepared using meal, while pellets are often used when dry feeding in order to optimise FE. However, pelleting can reduce particle size, and it is important that the proportion of particles <400 µm is minimised to prevent ulceration [53,54]. There are also distinct microbiological differences between feed forms, and their impact on pig gut microbial communities. For example, O’Meara et al. [39] found that LAB, *Enterobacteriaceae*, yeast, and mould counts were lower in a pelleted compared to a meal diet while Burns et al. [55] also found that *Enterobacteriaceae* counts were lower in pelleted feed than in meal (Table 2). This is likely due to the high temperatures and pressure used during the pelleting process. Mikkelsen et al. [56] also reported lower counts of total anaerobic bacteria, coliforms and yeast in pelleted compared to non-pelleted diets; however, there were no significant differences in LAB counts (Table 2).

Canibe et al. [57] reported higher levels of enterobacteria, yeast, and total aerobic bacteria in a coarsely ground meal diet compared to a finely ground pelleted diet (Table 2). Mikkelsen et al. [56] also investigated fine and coarse grinding of the feed and found that feeding coarsely ground meal to pigs reduced the pH and increased the lactic acid concentration in the gastric content, and consequently decreased the survival of *Salmonella* Typhimurium in the stomach [56]. A systematic review of the effect of feed characteristics and management practices on *Salmonella* prevalence in finisher pigs also associated non-pelleted diets with reduced *Salmonella* prevalence, albeit with a low degree of confidence [58]. Increased viscosity of the gastric content, and the associated slower gastric passage rate of coarsely ground meal is thought to facilitate the proliferation of LAB in the stomach and small intestine, along with the subsequent increase in lactic acid concentration and decrease in pH. This lower pH then exerts a barrier effect against pathogens i.e., conferring colonisation resistance [53,56,57,59].

It is also important to consider the impact of feed form on the microbial communities present in the pig GI tract. Interestingly, using quantitative polymerase chain reaction (qPCR), Lebel et al. [60] found that only *Bifidobacterium* (a genus used as a probiotic) was enriched in the faeces of pigs fed a meal diet and/or a diet with a large particle size [60]. Mikkelsen et al. [56] observed a decline in coliform counts in the distal small intestine, caecum and colon of pigs fed a coarsely ground as opposed to finely ground feed, with the lowest counts observed in pigs fed coarsely ground meal (Table 3). Canibe et al. [57] also found benefits in feeding a coarse meal diet, with higher counts of total anaerobic bacteria and LAB in the stomach and small intestine and lower enterobacteria counts in the caecum and colon compared to feeding a finely ground pelleted diet [57] (Table 3).

These studies indicate that coarsely ground meal is beneficial for gut health, facilitating the proliferation of LAB, production of lactic acid, and a subsequent decrease in pH, and inhibiting the growth of potentially pathogenic bacteria and their transit through the GI tract. These results, however, are in conflict with pig growth and nutritional data. Smaller feed particle size is known to improve pig growth due to increased digestibility, and pelleted diets (which usually have smaller particle sizes) are generally preferred due to the improved feed conversion ratio (FCR) observed [39,53,61]. Considering that meal has generally higher microbial counts compared to pellets, and that meal is the usual starting material for producing LF, it is reasonable to assume that its higher microbial load contributes to the spontaneous fermentation observed in LF. Differences in LF produced with meal or pellets may also impact on the gut microbiota of pigs fed these diets. The microbiota of liquid-fed pigs will be discussed in Section 6.

### 4.2. Fresh and Fermented Liquid Feed

**Figure 2:**
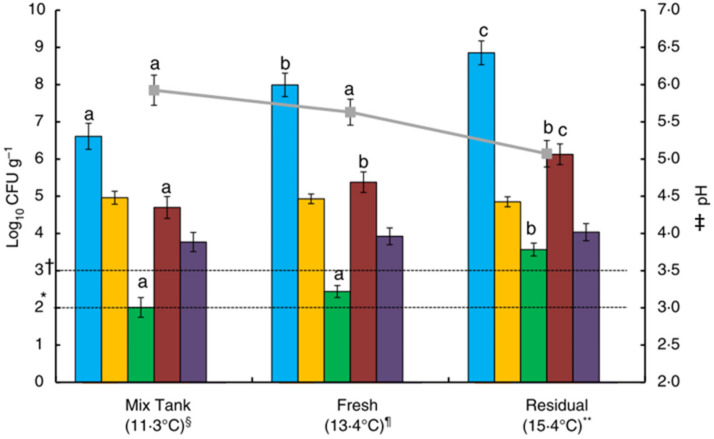
Mean (±SEM) counts of (blue) lactic acid bacteria; (orange) *Enterobacteriaceae*; (green) *Escherichia coli*; (brown) yeast and (purple) mould; and (grey line) feed pH ^‡^ in liquid feed samples from the mixing tank, fresh feed from troughs and residual feed from troughs on eight Irish commercial finisher pig units. * Detection limit for lactic acid bacteria, *Enterobacteriaceae* and *E. coli* (2 log_10_ CFU/g). ^†^ Detection limit for yeast and mould (3 log_10_ CFU/g). ^‡^ Sample pH is displayed on the secondary *y*-axis. ^§^ Mix Tank sample temperature = mean of data from eight samples. ^¶^ Fresh sample temperature = mean of data from 24 samples. ** Residual sample temperature = mean of data from 21 samples. ^a,b,c^ Within each bar colour and the line representing pH, bars and data points, respectively, that do not share a common letter are significantly different (*p* < 0.05). Adapted from O’Meara et al. [12].

As outlined earlier, the two main types of LF are fresh LF and FLF. The microbiology of FLF has been studied more extensively than that of fresh LF. However, due to the occurrence of unintentional spontaneous fermentation, it is likely that many farmers intending to feed fresh LF are in practice feeding a diet in which some degree of fermentation has occurred along the feed circuit. This is particularly the case with short trough *ad libitum* feeding, where feed remaining in troughs continues to ferment and acts as an inoculum for freshly delivered feed [12,16,50]. The degree of fermentation occurring in fresh LF is exemplified in a study by O’Meara et al. [12] which sampled finisher feed on commercial pig units feeding ‘fresh’ LF. They found that LAB, yeast and *E. coli* counts increased from the mixing tank to the residual feed remaining in the troughs, resulting in reduced pH, indicating the occurrence of spontaneous fermentation (Figure 2). The negative impact of this fermentation on the nutritional quality of the feed was evidenced by reduced levels of lysine, methionine, threonine and gross energy in the residual feed [12]. Canibe et al. [11] reported similar findings in fresh LF, with spontaneous fermentation evidenced by a loss of low molecular weight sugars and increases in LAB, yeasts and total anaerobes compared to dry feed, although they did not sample at different locations. High levels of *Enterobacteriaceae* and a pH of ~6 in the fresh LF, compared to dry feed and FLF, also indicated that the fresh LF was in the first phase of fermentation [11]. This spontaneous fermentation was likely accelerated by inoculation from residual feed remaining from the previous batch of feed. Additionally, the time lag between feeding and actual consumption of the feed likely contributed to the poor microbial quality, similar to the decline in the quality of residual feed in troughs reported by O’Meara et al. [12].

As a result of the potential for malfermentation and the subsequent issues with microbial and nutritional quality, different strategies have been employed to control and optimise the quality of LF for pigs. These range from simply backslopping (mixing fresh feed and liquid with a proportion of a previously successful fermentation as described in Section 1.2) to inoculation with a suitable LAB starter culture and can include whole diet fermentation, fermentation, or soaking of only the cereal component of the diet and/or enzyme supplementation. Physicochemical and microbiological data from studies examining the impact of these strategies on the microbial quality of LF are summarised in Table 4. Strategies are often combined. For example, Olstorpe et al. [21] investigated the effect of adding a four-strain LAB silage starter culture to cereal and wet wheat distillers’ grain in addition to 80% backslopping daily for 5 days, inoculating either at the start of the fermentation only, or at the start as well as daily at each backslopping. The latter appeared to be preferable as higher numbers of *Lactobacillus plantarum* (a component of the inoculant) were found; however, even with this approach, levels of acetic acid were high and lactic acid levels did not reach the desired concentration to completely exclude *Enterobacteriaceae* [21,62]. One reason for this was that the dominant organisms in the feed were *L. plantarum* (from the starter culture) and *Lactobacillus panis* (likely from the wet wheat distillers’ grain). Both of these species are heterofermentative and therefore likely responsible for the lower lactic acid and increased acetic acid production. Canibe et al. [36] surveyed ‘naturally produced’ FLF (where microbial inoculants were not used) on 40 Danish piglet farms. Despite LAB counts in the FLF being >8 log_10_ CFU/g, average lactic acid concentrations were ~90 mmol/kg, which is less than the desired concentration for successful fermentation (>150 mmol/L). This may be explained by the prevalence of four *Lactobacillus* phylotypes in the FLF, which were predominantly heterofermentative. Nonetheless, van Winsen et al. [15] achieved a lactic acid concentration of 261 ± 20 mmol/L with a *L. plantarum* starter (Table 4).

The use of a homofermentative LAB inoculant is preferable in order to maximise lactic acid production for pathogen inhibition and to minimise the production of undesirable metabolites from heterofermentative LAB. *Pediococcus pentosaceus*, a homofermentative LAB was present in the starter culture used in the study by Olstorpe et al. [21] but was only detected when the diet was inoculated daily, suggesting that conditions were not suitable for the strain to grow and produce lactic acid and that it was outcompeted by resident microbiota. This indicates that strains already adapted to LF conditions should be isolated and used as starter cultures [21].

Missotten et al. [64] screened a bank of LAB isolated from FLF and the porcine GI tract for their ability to produce good quality FLF (acetic acid <40 mmol/L, lactic acid >150 mmol/L, pH <4.5). Three strains of *Lactobacillus*; *Lactobacillus johnsonii*, *Lactobacillus salivarius* and *L. plantarum* were found to be very effective in the laboratory, meeting the organic acid and pH requirements and also exhibiting antimicrobial activity against *Salmonella* spp. [64]. Another strategy is to use a probiotic starter culture; for example, in two separate experiments, Missotten et al. [65] produced FLF with a commercial probiotic *Pediococcus acidilactici* (Bactocell^®^), with daily backslopping. However, at the end of both 28-day trials, *Lactobacillus* spp., not the probiotic *P. acidilactici*, dominated the fermentation, when the LF was either inoculated with the probiotic on days 0–2 and 24–26 or when the probiotic was added to the dry feed [65]. It is possible that the probiotic may have failed to dominate because the diet was fermented continuously with daily backslopping; therefore, batch fermentation may be a more suitable approach [14]. Nonetheless, fermenting LF with probiotic starter cultures may be an effective means of simultaneously improving the microbial quality of feed while delivering probiotics to the gut to improve pig health [20,66].

Although backslopping is a commonly used fermentation strategy, in practice, many farms feeding LF backslop unintentionally due to the impracticality of sanitising the liquid feeding system before a new batch of feed [16] (see Section 5). Moran et al. [67] found that for wheat fermentation, there was no advantage to increasing the backslopping proportion above 20% (up to 42% was trialed), in agreement with the findings of Dujardin et al. [18]. The 20% backslopping treatment reduced coliforms to the greatest extent, and yielded the highest lactate concentrations and the lowest pH. The authors also agreed with the consensus that high lactic acid concentrations and low pH are the key factors in coliform exclusion in LF. However, they noted that the time of exposure to these conditions is critical for coliform exclusion suggesting a minimum period of 24 h with a pH <4.0, coupled with high lactic acid concentrations [67], while Dujardin et al. [18] reported that coliforms were still not eliminated after 48 h under similar experimental conditions.

Fermentation of the cereal component of the complete diet (typically with LAB inoculants), followed by mixing with the remaining feed components prior to feeding has been used as a strategy, primarily to minimise the loss of synthetic amino acids due to microbial decarboxylation during the fermentation process [14,43]. Some other benefits of this strategy include a more rapid reduction in pH due to the lower buffering capacity of cereals compared to the whole diet [43,68]. A practical advantage is that a fermented cereal can be used as a component in multiple diets, as opposed to fermenting multiple whole diets separately, reducing the need for multiple fermentation tanks [14,67]. Torres-Pitarch et al. [33] compared fresh LF and LF in which the cereal fraction was fermented (fermented liquid cereal; FLC), both with and without carbohydrase supplementation. The cereal fraction was inoculated with a *L. plantarum*-*P. acidilactici* starter culture and fermented without adding or removing feed for an initial 52 h. This produced a fermented cereal fraction with desirable microbial characteristics: a pH of 3.7, LAB counts of 9.2 log_10_ CFU/g, undetectable *Enterobacteriaceae* and yeast counts of 6.8 log_10_ CFU/g. The use of high-throughput 16S ribosomal ribonucleic acid (rRNA) gene amplicon sequencing revealed the complete bacterial profile of a diet containing a fermented cereal fraction for the first time. Interestingly, both inoculated bacterial strains, although not specifically tracked during the fermentation, did not appear to predominate after the initial fermentation period, as *P. acidilactici* was undetectable after 52 h and *L. plantarum* was at a relative abundance of <2%. This was likely because the starter culture was a silage inoculant, highlighting the importance of selecting feed-specific starter cultures, as outlined above.

*Pantoea* and *Pseudomonas*, two potentially pathogenic bacterial genera, predominated in the dry cereal and at the start of the fermentation period. After the initial 52 h fermentation, LAB dominated the fermented cereal, with *Lactobacillus*, *Leuconostoc* and *Lactococcus* the most prevalent genera, in order of decreasing abundance. After the initial fermentation period, backslopping was performed and on day 10, *Lactobacillus* was still the predominant genus in both fermentation tanks (FLC and enzyme-supplemented FLC). However, by day 51, *Pediococcus parvulus* and several species of *Lactobacillus* dominated the FLC, but only *Lactobacillus* dominated the enzyme-supplemented FLC. The inability of *P. parvulus* to metabolise xylose (a sugar released by the xylanase present in the enzyme complex) was suggested to be the reason that *Pediococcus* did not dominate the enzyme-supplemented FLC [33]. Similar microbial profiles were observed in the mixing tanks, with a lower proportion of *Firmicutes* and a higher proportion of *Proteobacteria* in the fresh LF compared to the fermented cereal diets. At day 10, the mixing tanks of the enzyme-supplemented diets were dominated by *Lactobacillus* while the diets without enzyme supplementation were dominated by *Lactobacillus* and *Leuconostoc*. The microbial composition in the unsupplemented diets shifted at day 51 in that *Lactobacillus* and *Pediococcus* became the dominant genera, while *Lactobacillus* was still the dominant genus in the enzyme-supplemented diets, but at a higher relative abundance. However, the composition of the diets in the troughs, as opposed to the tanks, is most relevant as this is what the pigs consume. Interestingly, these showed the least differences among treatments, indicating that the fresh diets had undergone spontaneous fermentation, thereby becoming similar in microbial composition to the fermented cereal diets [33] (Table 4). As mentioned earlier, fermenting the cereal fraction of the diet is a strategy to minimise decarboxylation of synthetic amino acids. In fresh LF, lysine losses of >35% have been reported between the mixing tank and residual feed in troughs [12]. Lysine losses from the mixing tank to the troughs were <9% for the FLC diets, compared to 12% for the fresh LF diets in the study by Torres-Pitarch et al. [33], in agreement with other studies that also found lysine losses to be higher in FLF compared to FLC diets [43,68]. Canibe et al. [43] and Torres-Pitarch et al. [33] also detected lower levels of putrescine and cadaverine when the cereal fraction alone was fermented, with comparable concentrations across studies (Table 4), indicating that cereal fraction fermentation is a suitable strategy for minimising amino acid loss in LF.

In another study, Torres-Pitarch et al. [32] investigated the microbiota of LF where the cereal component of the diet (with or without a carbohydrase complex) was soaked for 3 h prior to mixing with the remaining dietary components immediately before feeding. Like the previous study, *Pseudomonas* and *Pantoea* were the predominant genera in the mixing tank, while in the troughs there was a shift to *Firmicutes*, primarily LAB including *Lactobacillus*, *Leuconostoc*, *Weisseilla* and *Lactococcus*, hence the high LAB counts obtained from the culture-dependent data (Table 4). This provides further evidence of spontaneous fermentation in residual LF remaining in short *ad libitum* troughs. There were less obvious differences in the microbiota between treatments; however, *Lactobacillus* was more abundant in all soaked diets, indicating that the enzyme complex released carbohydrates suitable for LAB fermentation during the enzyme soaking period. Additionally, similar to fermenting the cereal fraction of the diet alone, soaking the cereal fraction of the diet prior to mixing with the remainder of the diet resulted in lysine losses of <4% from the mixing tank to the troughs, while reductions of 12% were observed for the fresh LF diets, owing to the occurrence of spontaneous fermentation in the troughs [32].

Overall, the literature suggests that uncontrolled spontaneous fermentation of fresh LF contributes to decreased microbial and nutritional quality, with a need to implement strategies to improve feed quality. However, with deliberate fermentation, careful consideration should be given to the choice of microbial inoculant. The strain should be a homofermentative LAB isolated from feed and shown to be capable of dominating the fermentation and producing sufficient lactic acid. The inclusion of a suitable yeast strain that can dominate without contributing to off-flavours and significant energy loss in the diet should also be pursued. Cereal fraction fermentation or soaking also appear to be suitable strategies to improve the microbial and nutritional quality of LF, particularly in terms of minimising the loss of synthetic lysine. Nonetheless, analysis of the feed alone is not sufficient to justify use of these strategies. Section 6 will discuss how these strategies impact the pig gut microbiota, growth and FE.

### 4.3. Acidified Liquid Feed

One of the key benefits of fermenting LF is the increased production of organic acids from LAB fermentation, mainly lactic acid. Therefore, direct addition of organic acids to fresh LF is an alternative to intentional or spontaneous fermentation. As discussed, fermentation of LF can result in undesirable effects including decarboxylation of added synthetic amino acids, which has, at least in part, been attributed to *E. coli* and other members of the *Enterobacteriaceae* family [42]. Rapid acidification of LF may therefore minimise free amino acid loss as *Enterobacteriaceae* are at their highest levels during the first phase of fermentation, when the pH is high and levels of LAB (and thus lactic acid concentrations) are low [14]. Therefore, adding acid to LF during preparation should rapidly decrease the pH and inhibit the proliferation of enterobacteria. This approach has a similar aim to that of fermenting the cereal fraction of the diet prior to addition of synthetic amino acids, as discussed in the previous section.

Geary et al. [69] assessed the microbial profile of a fermented liquid weaner diet that was either supplemented with lactic acid or inoculated with *P. acidilactici* at the start of each new batch of feed. For the first 4 days of the experiment, coliforms increased in the *P. acidilactici*-inoculated FLF (pH 4.5) but then declined to become undetectable, while in the lactic acid-supplemented diet (pH 4) coliforms were eliminated after only 2 days. However, considering that both treatments were effective at inhibiting coliform growth, albeit at different rates, and there were no differences in the growth or FE of the pigs fed these diets, the authors suggested that fermentation with *P. acidilactici* is more practical. This is because to achieve the same drop in pH the cost of the inoculant was significantly lower compared to that of lactic acid. Lawlor et al. [63] also used lactic acid to acidify a liquid diet for weaned pigs. However, it should be noted that they prepared fresh diets daily, while Geary et al. [69] added lactic acid to each new batch of feed with residual feed from the previous batch remaining in the tank i.e., the diet was FLF supplemented with lactic acid. This difference in feed preparation was reflected by Lawlor et al. [63] reporting LAB counts ~2 log_10_ CFU/g lower than Geary et al. [69], along with lower yeast counts. However, Lawlor et al. [63] also reported higher coliform counts compared to the aforementioned study, which may have been a consequence of the lower LAB population.

Canibe et al. [17] performed an *in vitro* study to investigate acidification of a liquid grower diet with either formic acid or a commercial acid blend (Boliflor^®^ FA 2300S). The diets were fermented for 2 days with no feed or water removed or added, and thereafter the diets were backslopped with 10% retained after 48, 55, 72, 79, and 96 h of fermentation. The control diet showed a typical LAB fermentation pattern; after ~24 h LAB counts were ~8 log_10_ CFU/g, at which point the lactic acid concentration increased, the pH decreased and *Enterobacteriaceae* began to decline, although counts were still relatively high after day 2. In contrast, in the acidified diets, *Enterobacteriaceae* began to decline immediately due to the bactericidal effect of the acids already present, and LAB populations were much lower [17]. The authors also measured amino acid losses throughout fermentation. For all diets, at between 96 and 108 h of fermentation, losses were 26–34%, 31–38%, and 31–42% for free lysine, threonine, and methionine, respectively. Dietary acidification prior to fermentation was effective in reducing *Enterobacteriaceae* counts, albeit not to the desired extent, which likely contributed to this amino acid degradation. Unexpectedly, however, levels of putrescine, cadaverine, tyramine and histamine were below the detection limit of 10 mg/kg DM. The pH of the acidified diets rose during the first 48 h of fermentation, to ~5.5 in the case of Boliflor^®^ FA 2300S-supplemented feed. This may have been a factor in reducing the antibacterial activity of the organic acids as they are more effective at a lower pH i.e., the lower the external pH, the more undissociated acid can cross the cell membrane (depending on the pK_a_ of the individual acids) and reduce intracellular pH, ultimately leading to cell death [70]. Therefore, it is advisable to add acid to the diet to achieve a pH of 4 as performed by Lawlor et al. [63] and Geary et al. [69] with lactic acid. Further research is required to identify the microbes in LF that are responsible for amino acid degradation and to investigate the contribution of LAB [48,71].

Benzoic acid is another organic acid that has been used to control fermentation. It is used as a food and feed preservative, owing to its antibacterial and antifungal properties [72,73]. O’Meara et al. [74] examined the microbial quality of LF supplemented with benzoic acid (VevoVitall^®^) at inclusion levels of 0, 2.5, 5 and 10 kg/t. As in other studies, spontaneous fermentation of the control diet was observed in the troughs, as evidenced by reduced pH and higher LAB counts and ethanol concentrations compared to the mixing tank. Inclusion of 10 kg/t (1%) benzoic acid, however, appeared to control spontaneous fermentation to some extent, as evidenced by lower LAB counts and a higher pH in the troughs, compared to the control. However, yeast and *Enterobacteriaceae* counts were not affected by treatment, which like the previous study by Canibe et al. [17] may be due to an insufficient decrease in feed pH, albeit they did see reductions in *Enterobacteriaceae*. Interestingly, in the O’Meara et al. [74] study, the benzoic acid used did, however, appear to be more effective than the formic acid used by Canibe et al. [17] in preventing amino acid degradation, as the highest amino acid losses occurred in the control diet. In order to achieve a pH of 4 in the diet (so as to reduce *Enterobacteriaceae* effectively), the inclusion rate of benzoic acid would need to be 64.3 kg/t which would likely negatively affect growth and FE [75] in addition to being too costly. However, considering that many organic acids are either more effective for pH reduction or bactericidal activity, depending on their pK_a_ value and the pH of the feed, it is typically recommended to use a blend of acids in order to maximise both effects [76]. Although there appears to be some benefits associated with the addition of organic acids to LF, controlled microbial acidification with homofermentative strains may be a more appropriate and less expensive strategy to improve LF microbial quality. The impact on gut microbiota, growth and FE of ALF-fed pigs will be discussed in Section 6.2.

## 5. Liquid Feed System Sanitisation and Impact on the Microbial Profile of Liquid Feed

As discussed in Section 1.3, mixing of dry feed with water and/or co-products should lead to the development of a stable feed microbiota with desirable characteristics. However, malfermentation can occur resulting in undesirable metabolites, palatability issues, and loss of synthetic amino acids and energy [2,13,17,42]. This could suggest that greater attention should be paid to the sanitisation of liquid feeding systems to prevent carryover between feeds. However, it remains unclear whether routine cleaning and disinfection of liquid feeding systems is beneficial or detrimental to the quality of LF, and clear guidelines for farmers are lacking. Frequent cleaning of liquid feeding systems is generally not recommended, unless analysis of the LF has revealed that it is of poor microbial quality, in which case the system should be completely cleaned between batches of pigs using acids and/or bases [2,3,30,77]. Sanitisation practices used for liquid feeding systems on commercial farms are highly variable. For example, O’Meara et al. [12] surveyed cleaning practices on eight commercial pig farms in Ireland and found that mixing tanks were either not cleaned at all or were washed only with water, with the frequency of cleaning ranging from weekly to after every batch of pigs (Table 5).

In the Irish survey, cleaning of troughs varied from once or twice a year to after each batch of pigs. One farm used a detergent for cleaning troughs, one used lime after washing troughs with water, and only one farm used a disinfectant [12]. Despite the highly variable cleaning practices used on Irish farms, O’Meara et al. [12] found that these practices had no significant impact on microbial counts or pH of the LF, although a larger scale study is warranted.

**Figure 3:**
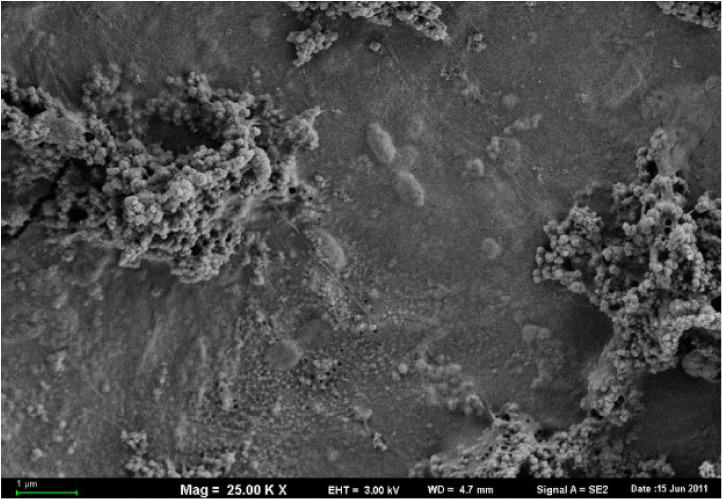
Scanning electron micrograph (×25,000 magnification) of bacterial cells in a biofilm (immersed in the extracellular polymeric substance matrix) on the surface of a high-density polyethylene pipe in a model drinking water distribution system (from Rozej et al. [81], distributed under the terms of the Creative Commons CC BY license).

The surface of the mixing tanks, feed lines and troughs of liquid feeding systems are coated with biofilms [78]. These are complex structures composed of a mucous-like extracellular polymeric matrix secreted by the enclosed microbes, which protects them from the environment [2,3,79] (Figure 3). In fact, recently, Heller et al. [78] found that administration of antibiotics via liquid feeding systems selects for antibiotic resistant bacteria within these biofilms that may potentially confer resistance to the feed microbiota and the pig gut microbiome. In a similar manner, biofilms in the pipelines may act as an inoculum for feed passing through the pipes and, therefore, the microbial community within the biofilm will likely affect the composition of the LF microbial community i.e., biofilms may act as a reservoir of LAB, yeast or potential pathogens. Biofilms are difficult to remove and are highly resistant to cleaning and, therefore, if the microbial composition of biofilms in the liquid feeding system is unfavourable, this may warrant intensive cleaning [80]. Conversely, if the pipeline is dominated by potentially beneficial LAB biofilms, cleaning may have a detrimental impact on feed quality. However, most likely due to the difficulty associated with sampling inside feedlines and mixing tanks, to our knowledge no studies to date have examined the impact of microbial biofilms on feed quality in LF systems.

Relatively few studies have explored the impact of cleaning on the microbiological quality of LF. The SEGES Danish Pig Research Centre reported that it took several days following cleaning and/or disinfection of liquid feeding systems for LAB fermentation to become re-established in the feed, allowing for the proliferation of coliforms in the days following cleaning, likely contributing to diarrhoea in the herds they studied [82]. They also recommended the addition of 2% formic acid to LF to control coliforms after cleaning. In addition, they highlighted that drop pipes in particular are of concern in terms of mould growth and mycotoxin contamination, and may require weekly high-pressure cleaning [82]. However, in a study conducted by Royer et al. [83] on nine finisher farms in France, four of which were termed ‘problematic’, with a high mortality rate associated with GI issues, no clear association between cleaning and disinfection of the feeding systems and pig mortality was made. The authors suggested that the origin of the problem was likely multi-factorial but was possibly related to the inconsistent impact of the cleaning/disinfection regime on the microbial quality of the LF [83].

Royer et al. [84] also performed a study to investigate the extent of microbial contamination in different parts of a liquid feeding system. They sampled ‘contact water’ that was passed through the system before and after an extensive cleaning and disinfection regime, to simulate the microbial load that a fresh batch of feed would acquire on passing through. Microbial counts, ATP readings (an indicator of hygiene) and pH measurements of contact water sampled before, immediately after, and 14 days after cleaning, at different points of the liquid feeding systems are presented in Table 6. They found that the microbial load increased from the mixing tank to the drop pipes. This is in agreement with O’Meara et al. [12], who showed that LAB and yeast counts increased in LF from the mixing tank to fresh feed in the troughs, with further increases in residual feed in the trough just prior to the next feed-out, coupled with decreased pH and higher organic acid concentrations, indicating the occurrence of spontaneous fermentation [12]. Hence, the drop pipes were identified as a concern [84], as was the case in the SEGES study outlined above [82]. The general trend found by Royer et al. [84] was that the cleaning and disinfection protocol resulted in a 2–3 log_10_ reduction in the bacterial load, but that within two weeks it had returned to pre-cleaning levels (Table 6). This possibly explains why O’Meara et al. [12] found no significant differences between farms that used different cleaning protocols. From this, Royer et al. [84] suggested that cleaning of the mixing tank alone will likely have little impact unless other parts of the system i.e., the drop pipes and troughs are also cleaned. They noted, however, that this extent of cleaning and disinfection may not be justifiable due to a lack of evidence linking LF system hygiene with a significant risk to pig health. However, recently another French group established a relationship between a decrease in the LAB:*Enterobacteriaceae* ratio (i.e., a deterioration of LF quality) and the occurrence of digestive problems including diarrhoea, torsion and oedema in liquid-fed finisher pigs across 49 farms [85].

The design of liquid feeding systems on individual farms must also be taken into account when considering sanitisation protocols. For example, in some feeding systems, particularly older ones, residual feed is allowed to sit in the feed lines between feeds (described as ‘residue-containing LF’ in Section 1.3). Brunon et al. [85] reported higher yeast and mould counts when residual LF remained stagnant in the feeding circuit compared to when pipes were rinsed with water and returned to a rinse tank, as is the case with ‘residue-free’ LF. It should be noted that for feeding systems where feed remains in the pipes between feeds, organic acids are often added to the feed to control fermentation in the pipes. Royer et al. [84] reported that in the case of rinse water remaining in the feeding circuit for several hours, natural acidification occurred but was not sufficient to inhibit coliforms. Nonetheless, coliform counts in the rinse water remaining in the pipes were reduced 14 days post-cleaning and disinfection [84], potentially due to disruption of biofilms. An alternative to rinse water is the use of high-pressure air to clean feed lines.

Residue-free liquid feeding can result in lower concentrations of organic acids and biogenic amines in LF [86]. Fisker and Jørgensen [30] suggested that extensive cleaning and disinfection of liquid feeding systems and regular weekly cleaning may have an effect similar to that of residue-free liquid feeding but by killing and/or inhibiting (rather than preventing proliferation of) undesirable microbes that produce fermentation products that can affect the pigs’ appetite and health. They investigated four groups of lactating sows receiving LF and experiencing issues with appetite, diarrhoea, low weaning weight and piglet mortality. Details of the cleaning and disinfection procedure used can be found in Fisker and Jørgensen [30] but, briefly, it involved filling, soaking, recirculating and rinsing the entire system with detergent and hot water (alkaline solution) followed by the same procedure with disinfectant (acidic solution). Liquid feed was sampled at 2-week intervals for 6 weeks before and 6 weeks after the cleaning and disinfection regime. No significant differences were observed in the pH, microbial counts and organic acid/biogenic amine concentrations between pre- and post-cleaning feed samples (Table 7). Therefore, the quality of the feed was unlikely to have been the cause of the sow health issues. However, the authors noted that the majority of the samples taken before cleaning were within the normal microbiological ranges for LF and this may explain why there was no significant difference in the quality of the feed following cleaning of the feeding system. They recommended that the entire system should be emptied and thoroughly cleaned if the *Enterobacteriaceae* count exceeds 7 log_10_ CFU/g in the feed. However, where *Enterobacteriaceae* counts are between 4 and 7 log_10_ CFU/g or if the mould counts are above 3 log_10_ CFU/g, the focus of cleaning should be on the mixing tank and supply pipes, not on emptying and cleaning the whole system [30].

In summary, studies investigating the impact of feed system sanitisation on the microbial quality of LF and/or the hygiene of feeding systems have yielded conflicting results, with more research required. However, in general, it does appear that intensive cleaning and disinfection of the entire system can improve the microbial quality of feed temporarily, but a quick return to pre-cleaning values occurs, with some studies showing no effect at all. In addition, immediately following cleaning, as at the beginning of fermentation, LAB counts are low, the pH is high and coliform blooms can occur in the LF [78,82], which may be mitigated by the use of microbial inoculants immediately after cleaning and/or by acidification of feed with organic acids. Farms should be assessed on an individual basis before implementation of any cleaning protocol. First of all, if the microbial quality of the feed is in question due to poor growth, appetite or health of the pigs, a review of the liquid feeding system should be carried out to eliminate any obvious issues.

The type of liquid feeding system must also be considered e.g., a residue-containing liquid feeding system where feed or rinse water is sitting in the pipes between feeds, may benefit from regular intensive cleaning. The microbial quality of feed prior to cleaning must also be assessed before implementing an intensive cleaning protocol, as these cleaning regimes are costly as well as being time- and labour-intensive. Therefore, if microbial quality parameters are within the normal range, there is likely no benefit to cleaning the feed system. Further research is warranted to investigate alternative cleaning protocols in addition to the composition of biofilms in liquid feeding systems and their influence on feed microbial quality and the harbouring of antibiotic resistance genes. Access to biofilms in feedlines is challenging; however, it may be possible to remove a small section of piping from the feeding circuit for analysis. Alternatively, studies on water distribution systems have used ‘test-plugs’ or disks which are mounted inside pipes and can be removed for analysis [79]. The literature also indicates that the drop pipes and troughs should be key areas for cleaning; firstly, due to the inflow of air to the drop pipes, promoting fungal growth, and secondly, if stale feed remains in the troughs it may reduce the quality of freshly delivered feed, even if the mixing tank and pipelines have been cleaned.

## 6. Impact of Liquid Feed on the Pig Gut Microbiome and Influence on Pig Growth Performance

Thus far we have discussed the microbiology of LF and ways to optimise microbial quality with the aim of improving gut health and growth of liquid-fed pigs. This section will discuss how LF and the aforementioned strategies to optimise feed microbial quality impact the gut microbiome of pigs, and their growth and FE. Research on the link between the pig gut microbiome, growth and FE has been growing in recent years, with some evidence of an association between taxa including *Lactobacillus* and *Ruminococcus*, for example, and growth and FE. However, there are conflicting reports and it can be difficult to determine cause and effect [87,88,89,90,91]. Nonetheless, liquid feeding may be an effective means of targeting these beneficial microbes. It is well established that one of the key benefits of feeding a fermented liquid diet to pigs, in terms of gut microbial ecology, is a reduction in pathogen numbers along the GI tract, provided that the pH and lactic acid concentration in the LF are at the desired levels [15,20,35]. Many of the studies that have examined the impact of different liquid feeding strategies on the GI tract of pigs have focused on key microbial groups via traditional culturing and molecular methods. However, an increasing number of studies are reporting high-throughput sequencing data, which is important in determining the full microbial profile of the GI tract of liquid-fed pigs and the impact of liquid feeding on these microbial communities.

### 6.1. Suckling and Weaned Pigs

Many liquid feeding studies have focused on suckling and weaned piglets. Liquid feeding of weaned pigs promotes higher feed intake, helping to avoid the post-weaning lag in growth normally observed after weaning, as well as facilitating the transition from sow’s milk to solid feed [20,92]. Additionally, during weaning, the ability of pigs to produce sufficient gastric acid is under-developed and, therefore, they depend on the fermentation of lactose to lactate in order to maintain a low pH in the stomach [14,93]. The stomach is the first line of defense against pathogens and therefore, the provision of LF with a low pH enhances this barrier effect. For these reasons, the benefits of LF tend to be more pronounced in suckling and weaned pigs compared to grow-finishers [13,20]. Liquid feeding, however, is often associated with poorer FE compared to dry feeding as it can lead to increased feed wastage and sedimentation of solids in the trough [16,50,51,94]. Improvements in trough design can however, minimise wastage and improve FCR [50].

Table 8 and Table 9 summarise the impact of various types of liquid feeding on the gut microbiota of suckling and weaned pigs, and the growth performance of piglets fed liquid diets, respectively. Mikkelsen et al. [34] fed naturally fermented FLF or fresh LF to weaners for a period of 4 weeks and found no differences in growth or FE between treatments, although average daily gain (ADG) and FCR were marginally improved for fresh LF. However, gastric pH and coliforms along the entire GI tract were lower in pigs fed FLF, while yeast counts were higher. Lawlor et al. [63] performed a series of experiments in pigs from weaning to slaughter, comparing growth performance after feeding fresh LF, lactic acid-ALF, FLF (*Lactococcus lactis*) or dry pellets for a period of 27 days, after which pigs were given dry pellets to 35 kg and a liquid finisher diet to slaughter. In the first two experiments, ADG and FE were poorer for fresh LF-fed pigs from weaning to 27 days post-weaning [92].

During the third and fourth experiments, pigs fed ALF had the highest ADG, while the dry pellet-fed pigs had the best FE. The authors suggested that feeding ALF may be beneficial for the first 27 days post-weaning; however, the benefit was not sustained beyond that period. On the other hand, Geary et al. [69] reported no growth benefit in pigs fed lactic acid-ALF for 28 days post-weaning. Nonetheless, other studies have shown much more promising results for liquid-fed weaned pigs [50,51,65,94,102]. Despite the improved growth rates often observed with liquid feeding, FCR is generally poorer than with dry feed, but there are exceptions, for example, a study by Missotten et al. [65] where sepiolite was added to a diet fermented with Bactocell^®^ to help prevent sedimentation of solids. Additionally, l’Anson et al. [102] found that soaking feed (i.e., mixing the dry feed with water and allowing it to steep for 1 h prior to feeding) improved feed intake, ADG and FCR compared to dry feeding weaners.

Van Winsen et al. [15] investigated the impact of *L. plantarum*-FLF on selected microbial groups of 10-week-old pigs challenged with *Salmonella* and found that FLF reduced *Enterobacteriaceae* counts along the entirety of the GI tract, with lower pH and higher *Lactobacillus* counts in the stomach. Similar findings were reported by Demecková et al. [97] who fed either *L. plantarum*-FLF, fresh LF or dry pellets to sows 2 weeks before farrowing, and for 3 weeks after. They found that coliform counts were lower, and LAB counts were higher in the faeces of sows fed FLF. Interestingly, coliforms were also lower in the faeces of piglets born to the sows fed FLF, while LAB counts were higher in piglets from sows fed either of the liquid diets compared to dry feed. Unfortunately, the growth of these piglets was not measured during the experimental period. The sow diet, microbiota and the microbes present in the environment (including the sow’s faeces) are known to be key influences on microbial colonisation of the piglet GI tract [104,105]; therefore, liquid feeding a diet of desirable microbial quality to sows prior to farrowing may be an effective means of promoting gut health of the offspring. Tajima et al. [95] fed weaned piglets *L. plantarum*-FLF and reported that the abundance of lactobacilli in the caecum decreased to ~30%; however, a number of beneficial butyrate-producing genera within the *Firmicutes* phylum including *Coprococcus*, *Roseburia* and *Faecalibacterium* increased in abundance and therefore improved caecal bacterial diversity.

Canibe et al. [43] fed weaners (28 days old) FLF or FLC (LF where the cereal fraction alone was fermented) and found no difference between feed intake or FE between treatments but ADG was increased for the pigs fed FLC 2 and 6 weeks post-weaning. Yeast counts along the GI tract of the pigs fed the FLC were consistently higher than when the whole diet was fermented. Additionally, terminal restriction fragment length polymorphism (TRFLP) analysis of digesta samples found fragments most likely representing *L. fermentum* to be dominant in the gut of pigs fed the FLF. This same fragment was also detected more frequently in the diet itself, and therefore it likely originated from the FLF. Similar to this, Pedersen et al. [96] found that heterofermentative lactobacilli increased in the faeces of weaned piglets fed fresh LF with wet wheat distillers’ grain compared to dry-fed and fresh LF-fed piglets, likely due to higher proportions of these strains in the wet wheat distillers’ grain. It should be noted that in the Canibe et al. [43] study, comparisons were not possible for dry-fed pigs because the fermented liquid diets were fed restrictively to avoid further fermentation in the troughs, while dry feed was fed *ad libitum*. The authors suggested that the restricted feeding of the liquid diets may have limited the growth potential of these pigs [43]. While in the Pedersen et al. [96] study, the pigs fed dry pelleted diets had improved growth and FE compared to those fed liquid diets, 5 weeks post-weaning, while the LF with wet wheat distillers’ grain reduced diarrhoea in the same period compared to the dry-fed and fresh LF-fed piglets [96].

Although growth performance was not investigated, He et al. [98] studied the impact of feeding a liquid diet fermented with a probiotic *Bacillus subtilis* strain to 7-day-old piglets for 25 days compared to feeding the same probiotic in a dry pelleted diet. Cruz-Ramos et al. [106] identified lactate, acetate, and 2,3-butanediol to be the main products of *B. subtilis* fermentation. Additionally, *B. subtilis* can secrete a range of extracellular carbohydrases and proteases to release simple sugars, organic acids and amino acids [107]. High-throughput 16S rRNA gene sequencing revealed that the pigs fed the *B. subtilis*-FLF had lower bacterial diversity but higher fungal diversity along the GI tract. The former may be a result of the influx of *Lactobacillus* into the GI tract of the pigs fed *B. subtilis*-FLF, while the latter may be a result of the introduction of LF-associated fungal taxa not normally resident in the GI tract. Whether these fungi colonise the gut or are only temporarily present is a matter of debate. The authors reported that during suckling and early post-weaning, *B. subtilis*-FLF promoted the growth of LAB; however, diarrhoea incidence was higher in this group, potentially due to the decreased bacterial diversity [98]. In the jejunum of pigs fed the *B. subtilis*-FLF the relative abundance of *Streptococcus*, *Clostridium sensu stricto*, *Bacteroides* and *Flavobacterium* was decreased, while in the colon, *Pseudobutyrivibrio*, *Lachnospiraceae*, *Erysipelotrichaceae*, *Ruminococcus* and *Clostridiales* were at higher relative abundances compared to pigs fed the pelleted diet. Elevated levels of primary and secondary bile acids were also found in the colon of pigs fed the *B. subtilis*-FLF and it was suggested that this was a result of an increase in bile acid-metabolising bacteria, which include some members of *Lachnospiraceae*, *Erysipelotrichaceae*, *Ruminococcus* and *Clostridiales* which were found in the colon. This may have contributed to increased diarrhoea in these piglets [98,108]. It is broadly known that bile acids are important for solubilising lipids and promoting their digestion and absorption [109]. These results indicate that further studies are required to better understand the effects of LF on intestinal microbiota composition and its potentially positive or negative health/production effects.

### 6.2. Grow-Finishing Pigs

Table 8 and Table 9 summarise studies that investigated the impact of various types of liquid feeding on the gut microbiota of grow-finisher pigs, and the growth performance of pigs fed liquid diets, respectively. DNA sequence-based information on the microbial profile of the GI tract of liquid-fed grow-finishers is still rather limited, although there are more data available compared to liquid-fed piglets. O’Meara et al. [39] investigated the optimal feed delivery method, as well as the optimal feed form for grow-finishers. They found that liquid-fed meal increased body weight and ADG, but worsened FE compared to feeding dry meal or wet/dry feeding meal [39]. Similar results were found for pigs fed a liquid pelleted diet compared to those fed dry and wet/dry-fed pelleted diets. Additionally, they found no advantage to liquid feeding a pelleted diet compared to a meal diet. The poorer FE of liquid-fed pigs was likely due to feed wastage as reported by others [50,51,94]. Additionally, the liquid diets in the O’Meara et al. [39] study were offered *ad libitum*, and considering that restricted feeding can improve FE compared to *ad libitum* feeding, this approach could mitigate against the negative effect on FE found in the study. However, restricted feeding will likely result in poorer growth as occurred in the study with weaned pigs by Canibe et al. [43]. Zoric et al. [101] found no differences between ADG, FE or carcass weight in pigs fed fresh LF compared to dry feed up until slaughter.

Canibe and Jensen [11] reported higher feed intake and ADG for growing pigs fed fresh LF compared to naturally fermented FLF and dry feed. However, similar to what has been observed in piglets fed FLF, the gastric pH and levels of enterobacteria along the GI tract of growing pigs fed FLF decreased compared to those fed fresh LF, while lactic acid concentrations were also numerically higher in FLF-fed pigs. Brooks et al. [99] compared fresh LF to three FLF diets that were inoculated with either *L. salivarius* (FLF-SAL), *P. acidilactici* (FLF-BAC), or a mixture of *P. acidilactici*, *P. pentosaceus*, *L. lactis*, and *L. plantarum* (FLF-STAB) to finisher pigs. There were no differences in ADG or FCR between treatments; however, coliform counts in the faeces of pigs fed the FLF-SAL diet were lower than in those fed fresh LF. Additionally, although LAB counts did not differ between treatments, the ratio of LAB:coliforms was higher in FLF-SAL and FLF-STAB compared to fresh LF.

Hurst et al. [4] fed grow-finishers fresh LF or lactic acid-ALF at different water:feed ratios using either restricted or *ad libitum* feeding compared to dry feeding. *Ad libitum* feeding of fresh LF (water:feed ratio of 3:1) improved ADG and lean tissue growth rate compared to *ad libitum* dry feeding. Both the fresh LF and lactic acid-ALF, when offered on a restricted basis, resulted in improved ADG, lower feed intake, and hence improved FE, compared to restricted dry feeding [4]. Braude et al. [103] also reported improvements in FE and ADG of growing pigs fed fresh LF, but at a water:feed ratio of 2.5:1. Benzoic acid has also been investigated as a means of improving growth in liquid-fed grow-finishers. Although 1% benzoic acid appeared to control spontaneous fermentation and minimise synthetic amino acid loss in LF [29,74], O’Meara et al. [74] found no impact on growth, FE or carcass quality as a result of dietary benzoic acid inclusion. However, management of the liquid feeding system was excellent compared to other studies and therefore, feed wastage was minimal even with *ad libitum* feeding, resulting in exceptionally good growth rates and FE for all treatments. The authors noted that this likely made it difficult to observe a significant improvement in these parameters in benzoic-acid supplemented pigs.

In another study by O’Meara et al. [68] fresh LF, FLF, LF with FLC and wet/dry feeding were compared in grow-finishers. In the first experiment, pigs fed the FLF (inoculated with Sweetsile which contains *L. plantarum* and *P. acidilactici*) had lower ADG and FE, and were lighter at slaughter than pigs on the other treatments, while feed intake was higher in pigs fed the fermented diets. Carcass quality measures were also poorer for the FLF-fed pigs. At slaughter, during the second experiment, pigs fed FLC weighed more than FLF- and wet/dry-fed pigs with similar weights to those fed fresh LF, while FE in wet/dry fed pigs was better than for FLF-fed pigs. Overall, fermenting the whole diet (FLF) resulted in poorer growth and FE, likely due to a loss of dietary energy as well as amino acid decarboxylation [68].

Although O’Meara et al. [39] did not investigate the gut microbiota of liquid-fed pigs in the aforementioned studies, Torres-Pitarch et al. examined the intestinal microbiota as well as the growth performance of grow-finishers fed fresh LF, FLF, or LF where the cereal fraction alone was either fermented i.e., FLC [33] or soaked [32], with or without enzyme supplementation. Fermenting the diet increased ADG and total tract nutrient digestibility, while supplementation with a xylanase and β-glucanase complex improved FE as well as ileal and total tract nutrient digestibility [33]. The gut microbiota composition of the pigs fed these diets provided an insight into why these improvements may have occurred. Beneficial bacteria correlated with improved growth, including *Lactobacillus kisonensis* and *Roseburia faecis*, were more abundant in the ileum and caecum of the enzyme-supplemented pigs, while fermentation of the cereal component of the diet decreased the abundance of gut bacteria including *Megasphaera* and *Streptococcus*, which were correlated with poorer growth. Considering that the inoculum strains did not dominate the feed, it is not surprising that they were not found in the gut of the pigs. However, interestingly, *P. parvulus* which was found to be dominant in the un-supplemented diets, was more abundant in the ileal digesta of pigs fed the unsupplemented-FLC diet.

Bunte et al. [100] fermented 60% of the whole diet, which was then supplemented with 40% non-fermented coarse cereals (referred to as partially fermented LF; PFLF). This was compared with fresh LF in one experiment and in a separate experiment fresh LF was compared to the fully fermented diet (FLF). There were no effects of either partial or whole diet fermentation on feed intake, body weight or FE, although both of the fermented diets marginally improved feed intake and FE compared to the fresh LF. The FLF, however, decreased pH and bacterial alpha diversity in the small intestine, but increased alpha diversity in the faeces [100]. The PFLF had a similar impact on bacterial alpha diversity compared to fresh LF (both diets in this experiment had phytase added) in that alpha diversity was decreased in the small intestine and increased further down the GI tract. Additionally, principal coordinate analysis (PCoA) of Bray–Curtis distances, in both experiments, demonstrated that the faecal microbiota of pigs fed the same diets clustered together and had higher dissimilarity to pigs fed the other diets. This was supported by permutational multivariate ANOVA of Bray–Curtis distances, which found that 23.7% of the variability in microbial composition was attributed to the diet. Beneficial taxa assigned to *Lactobacillus* and *Bifidobacterium* were at higher relative abundances in the GI tract of the pigs fed the PFLF. The authors suggested that supplementation of the diet with non-fermented coarse cereals (large particle size) may have promoted proliferation of these bacteria due to the presence of undigested carbohydrates in the large intestine that were then available for fermentation. As discussed in Section 4, coarse meal can also increase the viscosity and slow the passage of the GI content, facilitating the proliferation of LAB [100].

The study by Torres-Pitarch et al. that focused on cereal fraction soaking [32] was designed in a similar fashion to the fermentation experiment discussed earlier [33], except that the cereal fraction of the diets were soaked in water for 3 h instead of being fermented. Pigs fed the enzyme-supplemented soaked diet had higher ADG than pigs fed the enzyme-supplemented fresh LF (between day 0 and 21 of the experiment) with no other effects on ADG reported throughout the trial. Enzyme supplementation increased total tract nutrient digestibility; however, growth and FE were not improved. In contrast to the previous fermentation study by Torres-Pitarch et al. [33], cereal soaking and enzyme-supplementation appeared to reduce the relative abundance of taxa that were positively correlated with good performance parameters, while those that were negatively correlated with desirable traits/physiological measures were more abundant (Table 8). This may explain the limited improvement in growth and the lack of improvement in FE in pigs fed these diets. The intention of soaking the diet for 3 h was to improve nutrient digestibility by increasing the time the cereals are exposed to the enzyme complex. However, during the soaking period the feed was likely in the first phase of fermentation and, therefore, the less favourable gut microbiota and lack of growth and FE improvements may have been related to the prevalence of less favourable bacteria in the feed during this period. It should also be noted that these correlations should be interpreted with caution as they are merely associations between taxa and physiological data. Indeed, one of the biggest drawbacks of culture-independent methods, particularly using marker genes such as the 16S rRNA gene, is that taxa that have been associated with improved growth and FE often may not have been cultured previously, and therefore isolation from the pig GI tract is necessary to validate any *in vivo* claims. Equally, although compositional studies of the microbiota provide useful information, studies on the functionality of the microbiota using functional metagenomics and metabolomics, would provide greater insight as to how liquid feeding alters the functionality of the gut microbiome as opposed to identifying changes in the predominant taxa alone [59,90].

## 7. Conclusions

The negative impacts of spontaneous fermentation in fresh LF are evident from the literature, affecting both the microbial and nutritional quality of feed, resulting in poorer pig health and growth performance, which ultimately leads to economic losses for pig producers. However, if managed correctly, liquid feeding can be an effective strategy for improving pig growth and reducing feed costs, particularly if farmers have access to liquid co-products. Also, if finisher accommodation is limited on-farm, faster growth rates to reach a target slaughter weight can be achieved with liquid feeding. The strategies to improve the microbial and nutritional quality of LF discussed throughout this review provide opportunities to select for a desirable feed and gut microbiome to maintain the nutritional quality of the feed and to maximise the health and growth of liquid-fed pigs. However, there are some conflicting reports regarding the precise microbial taxa that play roles in improving growth and FE. The increasing availability and decreasing costs of shotgun sequencing and other omics technologies will enable us to gain useful insights into the functionality of the LF microbiome and the liquid-fed pig gut microbiome as opposed to relying on compositional data alone. However, even compositional culture-independent data from LF and liquid-fed pigs are limited, as mentioned earlier, particularly regarding the mycobiome.

There is also scope for improving LF microbial and nutritional quality through more practical approaches associated with cleaning and sanitisation of liquid feeding systems, which may limit spontaneous fermentation and its associated negative effects. Although the available data indicate that extensive cleaning may have short-lived benefits or no benefit at all, there is an opportunity to improve microbial quality by maintaining liquid feed system hygiene after cleaning. This may involve more frequent cleaning of the system after a less frequent intensive cleaning to limit a reversion to poor hygiene conditions, potentially with the use of organic acid blends. However, this will depend on the health status and performance of the pigs and the pre-cleaning microbial quality of the feed on a particular farm. Finally, the FE of liquid-fed pigs can be improved by better management of liquid feeding systems in order to reduce feed wastage; however, further improvements in FE can also be achieved using the aforementioned strategies to minimise the negative effects of uncontrolled fermentation, which include losses of amino acids and energy in the diet that could otherwise be utilised by pigs.

## Figures and Tables

**Table 1:** Standard pH values, microbiological parameters and organic acid levels in residue-free and residue-containing liquid feed [29,30].

Liquid Feed Characteristic/Component	Residue-Free Liquid Feed (Fresh LF) ^1^	Residue-Containing Liquid Feed (FLF) ^2^
pH	5.0–6.0	4.5–5.0
Lactic acid bacteria (CFU/g)	10^6^–10^8^	10^8^–10^9^
Yeast (CFU/g)	10^4^–10^6^	10^6^–10^7^
Enterobacteria (CFU/g)	10^4^–10^5^	<10^3^–10^4^
Fungi (CFU/g)	10^3^–10^4^	<10^3^
*Clostridium perfringens* (CFU/g)	<10^3^–10^4^	<10^2^
Lactic acid (mmol/kg)	0–10	40–150
Acetic acid (mmol/kg)	0–10	10–50
Formic acid (mmol/kg)	0–10	0–40
Ethanol (g/kg)	0–0.5	0.1–4

^1^ Residue-free liquid feed refers to fresh LF that is delivered to troughs immediately after the mixing of feed and water, following rinsing of the pipes. ^2^ Residue-containing liquid feed refers to fresh LF that is delivered to troughs immediately after the mixing of feed and water; however, feed from the previous batch remaining in the pipes acts as a fermentation inoculum (essentially producing FLF but differing from feed that is deliberately fermented).

**Table 2:** Microbiological data (log_10_ CFU/g) from experimental meal and pelleted diets (finely or coarsely ground) [39,56,57].

Reference	[39]		[56]				[57]	
Feed Form/Diet	Meal	Pellet	F-NP ^a^	F-P	C-NP	C-P	F-NP	C-NP
Lactic acid bacteria	3.30	2.29	3.29	3.31	3.76	3.42	<3.81 ^c^	<3.00
*Enterobacteriaceae*	5.24	3.26	NM	NM	NM	NM	<3.00	5.28
Yeast	3.92	3.12	4.33	4.43	3.30	3.14	<3.49	3.76
Mould	3.75	3.00	NM	NM	NM	NM	NM	NM
Total anaerobic bacteria	NM ^b^	NM	5.64	5.66	4.64	4.07	NM	NM
Total aerobic bacteria	NM	NM	NM	NM	NM	NM	4.74	6.08
Coliforms	NM	NM	5.10	4.88	3.45	3.14	NM	NM

^a^ F-NP = Fine non-pelleted diet; F-P = Fine pelleted diet; C-NP = Coarse non-pelleted diet; C-P = Coarse pelleted diet. ^b^ NM = Not measured. ^c^ The less than symbol (<) denotes that some observations from which the mean was calculated had values less than the detection level of 3 log_10_ CFU/g or had no colonies detected, in which case the detection limit was used as the value to calculate the mean.

**Table 3:** Microbiological counts (log_10_ CFU/g) in the gastrointestinal contents of pigs fed either meal or pelleted diets (finely or coarsely ground) [56,57].

	Reference	[56]				[57]	
GI Section	Microbial Group	Microbial Counts (Log_10_ CFU/g)					
		Diet ^a^					
		F-NP	F-P	C-NP	C-P	F-NP	C-NP
Stomach	Lactic acid bacteria	6.81	6.98	7.88	7.39	<6.6 ^c^	8.3
	Enterobacteria	NM ^b^	NM	NM	NM	<3.7	<3.7
	Coliforms	4.98	4.55	4.35	4.73	NM	NM
	Yeast	4.57	3.95	4.60	4.02	<4.1	<4.6
	Total anaerobic bacteria	7.06	7.16	8.53	7.59	<6.6	8.5
Distal small intestine	Lactic acid bacteria	7.89	8.42	8.50	8.31	8.4	8.9
	Enterobacteria	NM	NM	NM	NM	<6.3	<5.8
	Coliforms	6.65	6.46	6.01	6.05	NM	NM
	Yeast	5.54	4.89	5.20	5.12	<5.1	<5.3
	Total anaerobic bacteria	8.39	8.64	8.67	8.24	8.5	8.9
Caecum	Lactic acid bacteria	8.43	9.10	9.05	8.64	9.1	9.2
	Enterobacteria	NM	NM	NM	NM	7.1	6.4
	Coliforms	6.84	7.25	5.92	6.33	NM	NM
	Yeast	5.61	5.80	5.82	5.29	5.2	<5.2
	Total anaerobic bacteria	9.69	9.95	9.63	9.69	9.5	9.5
Mid-colon	Lactic acid bacteria	8.88	8.85	9.42	8.94	9.5	9.4
	Enterobacteria	NM	NM	NM	NM	6.8	<6.3
	Coliforms	7.53	6.44	5.87	6.45	NM	NM
	Yeast	5.39	4.65	5.58	5.48	5.2	<5.0
	Total anaerobic bacteria	9.66	10.22	9.91	9.91	9.9	<9.7

^a^ F-NP = Fine non-pelleted diet; F-P = Fine pelleted diet; C-NP = Coarse non-pelleted diet; C-P = Coarse pelleted diet. ^b^ NM = Not measured. ^c^ The less than symbol (<) denotes that some observations from which the mean was calculated had values less than the detection level of 3 log_10_ CFU/g or had no colonies detected, in which case the detection limit was used as the value to calculate the mean.

**Table 4:** Microbial and physicochemical properties of fresh, fermented, cereal fraction only fermented, soaked, acidified, and enzyme-supplemented liquid feed.

Type/Description of Liquid Feed [Microbial Inoculant if Applicable]	pH	Microbial Counts (log_10_ CFU/g)				Organic Acids, Biogenic Amines and Other Microbial Metabolites	References
		LAB	Enterobacteria /Coliforms ^h^	Yeast	Moulds		
FLC ^a^	5.00 ± 0.18	8.90 ± 0.33	<3.10 ± 0.30	7.80 ± 0.21	NM	Acetate: 13.00 ± 1.40 mmol/kg Lactate: 40.00 ± 5.70 mmol/kg Ethanol: 26.00 ± 4.10 mmol/kg Tyramine: 95.00 ± 13.00 mg/kg DM Putrescine: 75.00 ± 8.50 mg/kg DM Cadaverine: 153.00 ± 18.70 mg/kg DM Histamine: <11.00 ± 0.50 mg/kg DM	[43]
FLF ^b^	4.45 ± 0.11	9.30 ± 0.26	<3.50 ± 0.71	7.20 ± 0.24	NM	Acetate: 24.00 ± 2.40 mmol/kg Lactate: 160.00 ± 16.00 mmol/kg Ethanol: 17.00 ± 5.00 mmol/kg Tyramine: 40.00 ± 8.40 mg/kg DM Putrescine: 199.00 ± 123.30 mg/kg DM Cadaverine: 890.00 ± 151.30 mg/kg DM Histamine: 57.00 ± 2.20 mg/kg DM	
Fresh LF ^c^ (ENZ−) ^d^	4.50	8.50	5.50	6.60	<3.00	Acetate: 34.60 ppm Butyrate: 0.32 ppm Propionate: 0.42 ppm Cadaverine: 18.50 ppm Tyramine: <5.00 ppm Putrescine: 27.80 ppm Spermine: <5.00 ppm Spermidine: 34.90 ppm Histamine: <5.00 ppm	[33]
Fresh LF (ENZ+)	4.70	8.80	5.60	7.50	<3.00	Acetate: 39.30 ppm Butyrate: 0.40 ppm Propionate: 0.55 ppm Cadaverine: 19.30 ppm Tyramine: <5.00 ppm Putrescine: 62.10 ppm Spermine: 7.30 ppm Spermidine: 31.20 ppm Histamine: 36.00 ppm	
FLC (ENZ−) [*Lactobacillus plantarum* DSMZ16627 and *Pediococcus acidilactici* NCIMB3005]	4.50	9.10	5.10	7.20	<3.00	Acetate: 34.60 ppm Butyrate: 0.28 ppm Propionate: 0.56 ppm Cadaverine: 186.60 ppm Tyramine: <5.00 ppm Putrescine: 156.00 ppm Spermine: <5.00 ppm Spermidine: 31.60 ppm Histamine: 89.90 ppm	
FLC (ENZ+) [*Lactobacillus plantarum* DSMZ16627 and *Pediococcus acidilactici* NCIMB3005]	4.40	8.70	4.60	7.60	<3.00	Acetate: 65.40 ppm Butyrate: 0.32 ppm Propionate: 0.65 ppm Cadaverine: 6.50 ppm Tyramine: <5.00 ppm Putrescine: 20.70 ppm Spermine: <5.00 ppm Spermidine: 36.00 ppm Histamine: 86.10 ppm	
Fresh LF (ENZ−)	5.70	8.80	6.90	5.60	<3.00	Acetate: 22.50 ppm Butyrate: 0.30 ppm Propionate: 0.30 ppm Putrescine: <5 ppm Histamine: <5 ppm Cadaverine: 41.00 ppm Spermidine: 26.00 ppm Tyramine: <5 ppm Spermine: <5 ppm	[32]
Fresh LF (ENZ+)	5.00	8.70	6.90	6.10	<3.00	Acetate: 29.70 ppm Butyrate: 0.40 ppm Propionate: 0.80 ppm Putrescine: 6.00 ppm Histamine: <5.00 ppm Cadaverine: 89.00 ppm Spermidine: 8.00 ppm Tyramine: <5.00 ppm Spermine: <5.00 ppm	
SLC ^e^ (ENZ−)	5.70	8.90	6.40	6.60	<3.00	Acetate: 31.90 ppm Butyrate: 0.80 ppm Propionate: 0.40 ppm Putrescine: <5.00 ppm Histamine: <5.00 ppm Cadaverine: 46.00 ppm Spermidine: 13.00 ppm Tyramine: <5.00 ppm Spermine: <5.00 ppm	
SLC (ENZ+)	5.30	9.00	6.70	6.50	<3.00	Acetate: 28.60 ppm Butyrate: 0.30 ppm Propionate: 0.30 ppm Putrescine: 18.00 ppm Histamine: <5.00 ppm Cadaverine: 122.00 ppm Spermidine: 10.00 ppm Tyramine: <5.00 ppm Spermine: <5.00 ppm	
Fresh LF	5.98 ± 0.18	<6.90 ± 0.71	6.20 ± 0.59	5.00 ± 0.65	NM	Acetate: 2.30 ± 2.37 mmol/kg Lactic acid: 1.20 ± 2.31 mmol/kg	[11]
FLF	4.36 ± 0.17	9.40 ± 0.23	<3.20 ± 0.56	6.90 ± 0.69	NM	Acetate: 25.80 ± 6.32 mmol/kg Lactate: 168.60 ± 17.07 mmol/kg	
FLF	4.80	9.50 ± 0.34	6.70 ± 0.87	3.70 ± 0.95	NM	Acetate: 20.90 ± 6.21 mmol/kg Lactate: 91.20 ± 27.66 mmol/kg Butyric acid: 0.20 ± 0.07 mmol/kg Ethanol: 15.50 ± 1.31 mmol/kg Tyramine: <10.00 mg/kg DM Cadaverine: <10.00 mg/kg DM Putrescine: <10.00 mg/kg DM Histamine: <10.00 mg/kg DM	[17]
FLF (High feed intake group)	4.82	8.56	3.41	6.62	3.41	Acetate: 18.50 mmol/kg Lactate: 93.20 mmol/kg Ethanol: 18.90 mmol/kg Agmatine + Cadaverine + Histamine + Phenylethylamine + Putrescine + Spermidine + Tryptamine + Tyramine: 552 mg/kg DM	[36]
FLF (Low feed intake group)	4.70	8.45	3.26	6.14	3.45	Acetate: 17.70 mmol/kg Lactate: 90.50 mmol/kg Ethanol: 19.30 mmol/kg Agmatine + Cadaverine + Histamine + Phenylethylamine + Putrescine + Spermidine + Tryptamine + Tyramine: 528 mg/kg DM	
FLF [*Lactobacillus plantarum*]	4.10	NM	<DL	NM	NM	*L. plantarum*: 9.40 ± 0.26 log CFU/g *Salmonella* spp.: ND Lactate: 261.00 ± 20.00 mmol/L Acetate: 25.00 ± 13.00 mmol/L Butyrate: 2.30 ± 1.50 mmol/L Propionate and Ethanol: <DL	[15]
FLF (Diet 3) ^f^ [*Lactococcus lactis* subsp. *cremoris* 303]	4.14 ± 0.28	9.12 ± 0.23	1.33 ± 0.58 *	5.60 ± 0.26	3.84 ± 0.66	NM	[63]
FLF (Diet 4)	4.17 ± 0.32	9.39 ± 0.13	1.49 ± 0.56 *	5.60 ± 0.26	2.73 ± 0.60		
FLF (Diet 5)	4.10 ± 0.20	9.24 ± 0.03	< 1.00 *	7.07 ± 0.26	3.42 ± 0.57		
ALF ^g^ (Diet 3)	4.07 ± 0.17	5.44 ± 0.67	2.28 ± 1.15 *	4.2 ± 0.46	2.71 ± 0.63		
ALF (Diet 4)	4.09 ± 0.20	5.94 ± 1.30	1.55 ± 0.65 *	4.54 ± 0.84	2.32 ± 0.58		
ALF (Diet 5)	3.94 ± 0.28	6.39	<1.00 *	<1.00	5.40		
Dry feed (control)	4.80	9.50 ± 0.34	6.70 ± 0.87	3.70 ± 0.95	NM	Acetate: 20.9 ± 6.21 mmol/kg Lactate: 91.2 ± 27.66 mmol/kg Ethanol: 15.5 ± 1.31 mmol/kg Butyrate: 0.2 ± 0.07 mmol/kg Formic acid: <DL	[17]
ALF (Boliflor^®^ FA 2300S)	5.60	6.00 ± 0.63	3.30 ± 0.00	<3.00 ± 0.00	NM	Acetate: 4.9 ± 0.33 mmol/kg Lactate: <DL Ethanol: 1.7 ± 0.28 mmol/kg Butyrate: 0.4 ± 0.05 mmol/kg Formic acid: 34.2 ± 0.55 mmol/kg	
ALF (Formic acid)	5.30	<4.00 ± 0.00	5.20 ± 0.92	<3.30 ± 0.43	NM	Acetate: 4.6 ± 0.16 mmol/kg Lactate: <DL Ethanol: 0.4 ± 0.32 mmol/kg Butyrate: <DL Formic acid: 41.3 ± 0.67 mmol/kg	

^a^ FLC = Fermented liquid cereal: only the cereal fraction of the diet is fermented, with the remaining dietary ingredients added prior to feeding. ^b^ FLF = Fermented liquid feed: all dietary ingredients are fermented for a specific period of time prior to feeding. ^c^ Fresh LF = Fresh liquid feed: all dietary ingredients are mixed with water and fed out immediately. ^d^ ENZ = Xylanase and β-glucanase enzyme complex (Rovabio Excel AP, Adisseo France SAS, Antony, France). +/− indicates whether the diets were supplemented (+) or not (−) with ENZ. ^e^ SLC = Soaked liquid cereal: cereal fraction of the diet soaked in water for 3 h prior to mixing with balancer fraction (soybean meal, synthetic amino acids, minerals and vitamins) immediately prior to feeding. ^f^ Diets 3, 4 and 5 refer to different diets as per Table 1 in Lawlor et al. [63]. ^g^ ALF = Acidified liquid feed. ^h^ Either *Enterobacteriaceae* or coliform counts are reported. Coliform counts are denoted with an asterisk, counts without an asterisk represent *Enterobacteriaceae* counts. <DL = Less than detection limit. NM = Not measured. The less than symbol (<) denotes that some observations from which the mean was calculated had values less than the detection level of 3 log_10_ CFU/g or had no colonies detected, in which case the detection limit was used as the value to calculate the mean.

**Table 5:** Liquid feed system cleaning and sanitisation regimes and dietary co-product inclusion for finisher feed on eight Irish, and 27 Swiss pig farms.

References	[12]								[78]	
	Unit A	Unit B	Unit C	Unit D	Unit E	Unit F	Unit G	Unit H	Antibiotic (+) Farms (*n* = 13) ^A^	Antibiotic (−) Farms (*n* = 14)
Cleaning frequency MT *	2×/month ^†^	Never	Never	4×/day	1×/month	Never	1×/week	1×/10weeks	N/A	N/A
Cleaning agent MT	Water	N/A ^‡^	N/A	Formic acid ^§^	Water	N/A	Water	Water	N/A	N/A
Cleaning frequency pipes	2×/month	Never	Never	4×/day ^¶^	1×/month	Never	Never	4 to 5×/day	1×/6–10 days (*n* = 4) 1×/3 months (*n* = 13)	1×/week (*n* = 2) 1×/3 months (*n* = 5) 1×/6–12 months (*n* = 3) Never (*n* = 4)
Cleaning agent pipes	Water	N/A	N/A	Formic acid **	Water	N/A	N/A	Air ^††^	Organic acid (*n* = 4) ^B^ Caustic soda and/or sodium hypochlorite (*n* = 8) Never (*n* = 1)	Organic acid (*n* = 4) Caustic soda and/or sodium hypochlorite (*n* = 4) Other (*n* = 2) Never (*n* = 4)
Cleaning frequency troughs	Never	Never	Never	1×/~12weeks	2 to 3x/year ^‡‡^	Never	3×/year ^‡‡^	1×/10weeks	N/A	N/A
Cleaning agent troughs	N/A	N/A	N/A	Water	Water and lime ^§§^	N/A	Water and detergent ^¶¶^	Water and disinfectant ***	N/A	N/A
Co-product inclusion	Pot ale syrup (14%) and Liquid whey (21%)	N/A	N/A	N/A	N/A	Pot ale syrup (10%)	Pot ale syrup (15%)	N/A	Whey (*n* = 3) Acidified whey (*n* = 2 of 3)	Whey (*n* = 5) Acidified whey (*n* = 2 of 5)

* MT: Mix tank; ^†^ Twice per month; ^‡^ N/A: Not performed; ^§^ 85% Formic acid (Water Technology Limited, Cork, Ireland) at a 1% inclusion rate with water; ^¶^ 4x/day represents once after each feed daily; ** 85% Formic acid (Water Technology Limited, Cork, Ireland) at a 1% inclusion rate with water and mix sits in the feed pipes between feeds; ^††^ Hydro Air liquid feeding system; ^‡‡^ Except during the winter months; ^§§^ Water followed by lime; ^¶¶^ Water followed by detergent (Top Foam^™^, MS Schippers, Bladel, The Netherlands); *** Water followed by disinfectant (Hyperox, Du Pont, Sudbury, United Kingdom). ^A^ Antibiotic (+) farms were administering in-feed antibiotics to finisher pigs. Antibiotic (−) farms had not administered in-feed antibiotics for at least 2 years. ^B^ Cleaning agents for pipes for Heller et al. [78] were either added to water for circuit pipeline cleaning or flushing after cleaning.

**Table 6:** Microbiological counts (log_10_ CFU/g), ATP readings (relative light units) and pH (± SD) of contact water sampled at different locations on nine pig farms before, immediately after and 14 days after extensive cleaning of the liquid feeding systems (adapted from Royer et al. [84]).

Microbial Groups and Other Parameters	Group		Time of Sampling ^b^			Sampling Location ^c^		
	Pb+ ^a^ (*n* = 36)	Pb− (*n* = 45)	Before (*n* = 27)	After (*n* = 27)	D14 (*n* = 27)	Mixing Tank (*n* = 27)	Main Circuit (*n* = 27)	Drop Pipes (*n* = 27)
**pH (*n* = 81)**	7.35 ± 0.46	7.18 ± 0.62	7.05 ± 0.59	7.66 ± 0.42	7.07 ± 0.42	7.31 ± 0.46	7.34 ± 0.48	7.13 ± 0.69
**ATP (*n* = 81)**	3.54 ± 0.73	3.67 ± 0.86	4.03 ± 0.76	3.23 ± 0.54	3.55 ± 0.88	2.90 ± 0.61	3.60 ± 0.48	4.31 ± 0.61
**Total bacteria (*n* = 79)**	4.79 ± 1.63	4.70 ± 2.18	6.04 ± 1.60	3.06 ± 1.61	5.19 ± 1.35	3.56 ± 1.67	4.75 ± 1.90	5.86 ± 1.61
**Lactic acid bacteria (*n* = 79)**	4.16 ± 1.82	4.00 ± 2.28	5.28 ± 1.68	2.29 ± 1.67	4.71 ± 1.60	2.91 ± 1.80	4.03 ± 1.90	5.30 ± 1.89
**Coliforms (*n* = 80)**	2.13 ± 1.27	2.19 ± 1.31	2.70 ± 1.32	1.24 ± 0.85	2.55 ± 1.22	1.57 ± 1.07	2.09 ± 1.17	2.83 ± 1.30

^a^ Pb+ and Pb− groups are four and five problematic and non-problematic farms in southwestern France, respectively. Pb+ refers to a mortality rate associated with gastrointestinal issues >4% during the finisher stage, while Pb− farms did not have issues with mortality. ^b^ Time of sampling: values before cleaning (Before), immediately after cleaning (After), and 14 days after cleaning (D14) for Pb+ and Pb− farms. ^c^ Sampling location: samples of rinse water taken from the mixing tank where the diets are prepared (mixing tank), from lines of the main circuit (main circuit), and from drop pipes above the troughs (drop pipes). Values are means of all sampling times (before, immediately after, and 14 days after cleaning for Pb+ and Pb− farms).

**Table 7:** Microbiological counts and organic acid and biogenic amine concentrations in liquid feed sampled from valves mid-way along the circuit of four farms before and after cleaning and disinfection of the entire liquid feeding system (adapted from Fisker and Jørgensen [30]).

		Microbial Counts (log_10_ CFU/g) and pH							
Farm No.		pH	Enterobacteria	Lactic Acid Bacteria	Yeast	Mould	*Clostridium perfringens*		
1	Before ^a^	4.6	<3.5 ^c^	9.1	7.0	<3.1		<2.2	
	After	4.5	<3.5	9.0	6.6	<3.0		<2.4	
2	Before	4.8	<3.7	8.9	6.6	<3.0		<2.0	
	After	5.0	4.0	9.0	6.7	<3.0		<2.0	
3	Before	4.9	5.4	9.1	6.0	<3.0		<2.0	
	After	5.0	4.9	9.1	6.5	<3.0		<2.0	
4	Before	5.1	4.9	8.9	6.5	<3.8		<2.0	
	After	4.9	<3.2	9.0	6.6	<3.0		<2.0	
		**Organic acids and ethanol (mmol/kg)**							
		**Formic** **Acid**	**Acetic** **Acid**	**Propionic** **Acid**	**Lactic** **Acid**	**Succinic** **Acid**	**Butyric** **Acid**	**Ethanol**	
1	Before	21.1	15.3	3.9	81.5	1.5	ND	21.0	
	After	20.8	12.2	1.4	81.7	ND	2.7	16.7	
2	Before	9.1	18.4	ND	103.7	ND	0.6	22.4	
	After	4.2	14.5	ND	83.1	ND	0.6	15.3	
3	Before	2.8	12.6	4.0	88.4	1.2	2.3	6.4	
	After	ND ^b^	13.8	1.1	81.7	1.1	1.4	13.0	
4	Before	ND	10.8	0.5	56.2	0.5	4.4	8.7	
	After	5.3	14.2	1.6	82.6	ND	1.2	14.4	
		**Biogenic Amines ^d^ (mg/kg dry matter)**							
		**Phe**	**Cad**	**His**	**Put**	**Spd**	**Spr**	**Try**	**Tyr**
1	Before	1	729	5	115	54	19	118	4
	After	1	983	2	172	61	17	136	3
2	Before	1	564	35	127	48	17	64	4
	After	1	811	44	129	50	20	10	4
3	Before	1	126	30	44	47	20	13	4
	After	1	267	44	64	47	20	40	4
4	Before	1	582	23	90	46	17	6	2
	After	1	471	20	67	43	14	18	3

^a^ Before = Mean of values from liquid feed sampled at 2-week intervals for 6 weeks before the cleaning and disinfection regime. After = Mean of values from liquid feed sampled at 2-week intervals for 6 weeks after the cleaning and disinfection regime. For each farm, three samples were taken before (for farm 1, four samples were taken before cleaning) and three samples after the liquid feeding system had been cleaned and disinfected. All samples were taken during a normal feeding from a valve in the middle of the feeding circuit. ^b^ ND = Not detected. ^c^ The less than symbol (<) denotes that one or more observations from which the mean was calculated had values less than the detection limit (3 log_10_ CFU/g for enterobacteria and mould; 2 log_10_ CFU/g for *Clostridium perfringens*). ^d^ Phe = phenylethylamine; Cad = cadaverine; His = histamine; Put = putrescine; Spd = spermidine; Spr = spermine; Try = tryptamine; Tyr = tyramine.

**Table 8:** The impact of feeding fresh, fermented, cereal fraction only fermented, soaked and enzyme-supplemented liquid feed on the gut microbiota of suckling, weaned and grow-finishing pigs.

Details of Pigs, Initial Age/Weight If Applicable (No. of Pigs)	Type of Liquid Feed	Methodology	GI Section/Sample Type (No. of Samples)	Differences in Gut Microbial Taxa/Diversity (and pH if Applicable) between Treatments	Reference
**Suckling and weaned pigs**					
28 days/7.9 ± 1.0 kg (*n* = 30)	FLF ^a^ (Bactocell^®^ added to water and feed)	Plate counts	Faeces (*n* = 3)	NSD ^j^ between coliform and *Lactobacillus* counts between pigs fed FLF or dry diet	[65]
27 days/6.3 ± 1.2 kg (*n* = 45)	FLF (Bactocell^®^ already added to dry diet before mixing)			NSD between coliform and *Lactobacillus* counts between pigs fed FLF, dry diet or dry diet with Bactocell^®^, except ↓ *Lactobacillus* spp. in FLF vs. dry diet with Bactocell^®^ (day 8 of the trial)	
24 days/6.98 ± 0.15 kg (*n* = 360)	Fresh LF ^b^	qPCR ^h^	Caecum (*n* = 6)	↑ total bacteria vs. dry feed	[92]
28 days (*n* = 25)	FLF (*Lactobacillus plantarum* LQ80)	16S rRNA gene cloning and sequencing	Ileum (*n* = 3)	↓ *Sarcina* vs. dry feed	[95]
			Caecum (*n* = 3)	↑ *Dorea*, Lachnospiraceae *Incertae Sedis* vs. dry feed ↓ *Lactobacillus* vs. dry feed	
28 ± 1 days/8 ± 1.1 kg (*n* = 120)	FLF	Plate counts	Stomach (*n* = 8)	↑ LAB ^k^ (20 °C) vs. dry, ↓ yeast (37 °C) vs. FLC, ↓ yeast (20 °C) vs. FLC, ↑ yeast (20 °C) vs. dry	[43]
			Caudal small intestine (*n* = 8)	↓ LAB (37 °C), ↑ LAB (20 °C), ↑ yeast (20 °C) vs. dry, ↓ yeast (37 °C) vs. FLC	
			Caecum (*n* = 8)	↓ yeast (37 °C) vs. FLC and dry	
			Mid-colon (*n* = 8)	↑ LAB (37 °C), ↑ LAB (20 °C) vs. dry, ↓ yeast (37 °C) vs. FLC	
	FLC ^c^		Stomach (*n* = 8)	↑ LAB (20 °C) vs. dry, ↑ yeast (37 °C and 20 °C) vs. FLF and dry	
			Caudal small intestine (*n* = 8)	↑ LAB (20 °C), ↑ yeast (20 °C) vs. dry, ↑ yeast (37 °C) vs. FLF and dry	
			Caecum (*n* = 8)	↑ yeast (37 °C), ↑ yeast (20 °C) vs. FLF	
			Mid-colon (*n* = 8)	↑ yeast (37 °C) vs. FLF, ↓ LAB (37 °C) vs. dry	
28 days/7.9 ± 1.1 (*n* = 20)	Fresh LF	Plate counts	Stomach (*n* = 10)	↑ pH vs. FLF	[34]
			Small intestine (*n* = 10)	↓ pH vs. FLF	
			Entire tract (*n* = 10)	↓ yeast, ↑ coliforms vs. FLF	
34.3 ± 2.2 days/9.1 ± 1.7 kg (*n* = 48)	Fresh LF vs. fresh LF (+ WWDG ^d^) vs. dry	Plate counts and 16S rRNA gene sequencing of individual isolates	Faeces (*n* = 4)	NSD in diversity of coliforms or lactobacilli between diets. Similar composition of lactobacilli on day 1. On day 36, pigs fed fresh LF (+WWDG) were dominated by obligate heterofermentative lactobacilli (74%) vs. 35% and 49% for dry and fresh LF, respectively	[96]
Farrowing sows (*n* = 18) and their piglets	FLF (*Lactobacillus plantarum)*	Plate counts	Faeces (NR)	↓ coliforms in sows fed FLF vs. fresh LF and dry (7 days after parturition) ↓ LAB in sows fed fresh LF and dry after farrowing ↑ LAB in piglets (7 days old) when sows were fed FLF and fresh LF vs. dry ↓ coliforms in piglets (7 days old) when sows were fed FLF vs. fresh LF and dry	[97]
7 days/~2.8 kg (*n* = 110)	FLF (*Bacillus subtilis*) vs. dry pellets (*Bacillus subtilis*)	Pyrosequencing of V1–V3 region of the 16S rRNA gene	Jejunum (*n* = 6)	↓ Observed OTUs ^l^, Chao1 and Shannon diversity in FLF vs. dry pellets ↓ *Streptococcus*, *Clostridium sensu stricto*, *Bacteroides* and *Flavobacterium* in FLF vs. dry pellets	[98]
			Colon (*n* = 6)	↑ Observed OTUs, Chao1 in FLF vs. dry pellets ↑ *Pseudobutyrivibrio*, *Lachnospiraceae*_*unclassified*, *Erysipelotrichaceae*_*unclassified*, *Ruminococcus*, *Clostridiales*_*unclassified* and *Lachnospiraceae*_*uncultured* in FLF vs. dry pellets	
10 weeks (*n* = 48)	FLF (*Lactobacillus plantarum*) vs. dry feed in *Salmonella*-challenged pigs	Plate counts	Stomach (*n* = 10)	↓ pH, ↑ lactobacilli, ↓ *Enterobacteriaceae*	[15]
			Ileum (*n* = 10)	↓ *Lactobacillus plantarum*, ↓ *Enterobacteriaceae*	
			Caecum (*n* = 10)	↓ *Enterobacteriaceae*	
			Colon (*n* = 10)	↓ *Enterobacteriaceae*	
			Rectum (*n* = 10)	↑ pH, ↓ *Enterobacteriaceae*	
**Grow-finisher pigs**					
31 ± 3.5 kg (*n* = 60)	FLF vs. fresh LF vs. dry	Plate counts	Stomach (*n* = 5)	↓ pH, ↓ total anaerobes, ↑ LAB (20°C), ↓ enterobacteria, ↑ yeast (20°C and 37°C) for FLF vs. fresh LF and dry	[11]
			Mid-small intestine (*n* = 5)	↓ pH for fresh LF vs. FLF and dry	
			Distal small intestine (*n* = 5)	↓ pH for fresh LF vs. dry, ↑ LAB (20 °C), ↓ enterobacteria for FLF, ↑ yeast (20 °C) vs. fresh LF and dry	
			Caecum (*n* = 5)	↓ LAB (37 °C) in FLF vs. fresh LF, ↓ enterobacteria in FLF vs. fresh LF and dry	
			Mid-colon (*n* = 5)	↓ total anaerobes, ↓ LAB (37 °C), ↓ enterobacteria for FLF, ↑ yeast (20 °C) vs. fresh LF and dry	
			Distal colon (*n* = 5)	↓ pH for fresh LF vs. FLF	
16 weeks/54.3 ± 6.3 kg (*n* = 32)	FLF (*Lactobacillus salivarius*) vs. FLF (Bactocell^®^) vs. FLF (Stabisil^™^) vs. fresh LF	Plate counts	Faeces (*n* = 8)	↓ coliforms for FLF (Stabisil^™^) vs. other treatments, NSD in LAB counts between treatments, ↑ LAB:coliform ratio of FLF (*Lactobacillus salivarius*) and FLF (Stabisil^™^) vs. fresh LF and FLF (Bactocell^®^)	[99]
33.4 ± 0.88kg (*n* = 392)	Fresh LF vs. fresh LF + ENZ ^e^ vs SLC ^f^ vs SLC + ENZ	250 bp ^i^ paired-end Illumina amplicon sequencing of V3-V4 region of the 16S rRNA gene	Ileum (*n* = 6)	↑ *Cellulolysiticum* (negatively correlated with ADG ^m^) in SLC + ENZ vs. SLC ↑ *Leuconostoc mesenteroides, Lactococcus* and *Lactococcus raffinolactis* (taxa negatively correlated with carcass weight) in SLC + ENZ vs. fresh LF + ENZ	[32]
			Caecum (*n* = 6)	↓ *Megasphaera elsdenni* (positively correlated with carcass weight and butyrate) in fresh LF + ENZ vs. fresh LF ↑ Prevotellaceae NK3B31 sp., *Oscillibacter* sp. and *Rikenellaceaceae_RC9* (negatively correlated with growth parameters and butyrate) in fresh LF + ENZ vs. fresh LF ↓ *Selenomonas* (positively correlated with ADG) in SLC + ENZ vs. SLC ↑ *Escherichia/Shigella/Brenneria* (negatively correlated with ADG) in SLC + ENZ vs. fresh LF + ENZ ↑ *Prevotellaceae_NKB31_group* sp. and *Clostridium saudiense*/*disporicum* (negatively correlated with carcass weight, ADG, and butyrate) in SLC + ENZ vs. SLC ↓ *Roseburia faecis*/*intestinalis*/*hominis* (positively correlated with ADG and butyrate) in soaked diets vs. fresh diets ↑ *Rikenellaceae_RC9* (positively correlated with butyrate concentration) in soaked diets vs. fresh diets	
40.6 ± 0.50 kg (*n* = 252)	Fresh LF vs fresh LF + ENZ vs FLC (Sweetsile^®^) vs FLC + ENZ (Sweetsile^®^)	250 bp paired-end Illumina amplicon sequencing of V3-V4 region of the 16S rRNA gene	Ileum (*n* = 6)	↑ *Pediococcus* in FLC (17% relative abundance vs. 1–3% for other treatments ↑ *Lactobacillus kisonensis* in fresh LF + ENZ, FLC and FLC + ENZ (positively correlated with ADG) ↓ *Megasphaera* (negatively correlated with carcass weight) in fresh LF + ENZ ↑ *Bifidobacterium* (negatively correlated with ADG) and *Howardella* (positively correlated with butyrate and negatively correlated with ADG) in fresh LF + ENZ ↑ *Streptococcus* (negatively correlated with ADG) in FLC + ENZ ↓*Megasphaera*, *Bifidobacterium*, *Streptococcus*, *Howardella* and *Streptococcus pasteurianus*/*alactolyticus*/*macedonicus* (negatively correlated with either carcass weight or growth) in fermented cereal diets	[33]
			Caecum (*n* = 6)	↑ pH for FLC + ENZ vs. other treatments ↑ *Roseburia faecis* in LF + ENZ and FLC + ENZ (positively correlated with ADG)	
61 ± 2 days/20.8 ± 2.06 kg (*n* = 20)	FLF (*Lactobacillus plantarum*, *Pediococcus pentosaceus* and *Lactococcus lactis*) vs. fresh LF	Amplicon sequencing of the V4 region of the 16S rRNA gene	Small intestine (*n* = 20)	↓ pH, ↓ Shannon diversity in pigs fed FLF vs. fresh LF	[100]
			Colon (*n* = 20)	↑ pH, Numeric ↑ in Shannon diversity in FLF vs. fresh LF (NSD)	
			Faeces (*n* = 20)	↑ Observed species, Chao1 and Shannon diversity in FLF vs. fresh LF	
	PFLF ^g^ (with non-fermented coarse cereals) vs. fresh LF		Small intestine (*n* = 20)	↑ *Lactobacillus*, ↑ *Bifidobacterium*, ↓ pH, ↓ Observed species, Chao1 and Shannon diversity in PFLF vs. fresh LF, ↑ *Leuconostoc* in fresh LF vs. PFLF	
			Colon (*n* = 20)	↑ *Lactobacillus*, ↑ *Bifidobacterium*, numeric ↑ in Shannon diversity in PFLF vs. fresh LF (NSD), ↑ *Leuconostoc* in fresh LF vs. PFLF	
			Faeces (*n* = 20)	↑ *Lactobacillus*, ↑ *Bifidobacterium*, ↑ Shannon diversity in PFLF vs. fresh LF	

^a^ FLF = Fermented liquid feed: all dietary ingredients are fermented for a specific period of time prior to feeding. ^b^ Fresh LF = Fresh liquid feed: all dietary ingredients are mixed with water and fed out immediately. ^c^ FLC = Fermented liquid cereal: only the cereal fraction of the diet is fermented, with the remaining dietary ingredients added prior to feeding. ^d^ WWDG = Wet wheat distillers’ grain. ^e^ ENZ = Xylanase and β-glucanase enzyme complex (Rovabio Excel AP, Adisseo France SAS, Antony, France); +/− indicates whether diets were supplemented (+) or not (−) with ENZ. ^f^ SLC = Soaked liquid cereal: cereal fraction of the diet soaked in water for 3 h prior to mixing with balancer fraction (soybean meal, synthetic amino acids, minerals and vitamins) immediately prior to feeding. ^g^ PFLF = Partially fermented liquid feed: rapeseed extracted meal and part of the rye (60% of the whole diet) were fermented while the remaining cereal components were not fermented. ^h^ qPCR = Quantitative polymerase chain reaction. ^i^ bp = Base pairs. ^j^ NSD = No statistical difference. ^k^ LAB = Lactic acid bacteria. ^l^ OTUs = Operational taxonomic units. ^m^ ADG = Average daily gain.

**Table 9:** Growth performance, feed intake, feed efficiency and carcass quality of suckling, weaned and grow-finishing pigs fed fresh, fermented, cereal fraction only fermented, soaked and enzyme-supplemented liquid feed.

Initial Age/Weight of Pigs (Number of Pigs)	Type of Liquid Feed	Water: Feed Ratio	Performance ^i^	Notes	Reference
**Suckling and weaned pigs**					
24 days/6.98 ± 0.15 kg (*n* = 360)	Fresh LF ^a^	4:1	↑ BW, ↑ ADFI, ↑ ADG, same G:F vs. dry meal	Piglets weaned at 24 ± 1 days fed fresh LF for 7 days	[92]
32 days/19.3 kg (*n* = 280)	Fresh LF	2.15–2.23:1	Similar ADG, FCE and carcass weight vs. dry feed	Piglets were weaned at 32 days with access to dry creep feed from 10 days of age until the trial began. Water:feed ratio of fresh LF was 2.15: 1 (up to 70 kg LW) and 2.23: 1 (from 70–115 kg LW)	[101]
26 days/8.4 kg (*n* = 192)	Fresh LF	2:1	↑ DMI, ↓ G:F vs. dry pellets (d 0 to slaughter)	Length of each experiment was 27 days. After day 27 pigs were given dry pellets to 35 kg and liquid finisher diet (3:1 water:meal) to slaughter (95 kg)	[63]
26 days/8.4 kg (*n* = 150)	Fresh LF		↓ LW and carcass weight, ↓ G:F, ↑ lean meat % vs. dry pellets (d 0 to slaughter)		
26 days vs. 7.7 kg (*n* = 112)	Fresh LF		NSD in performance vs. dry pellets and ALF (d 0 to slaughter)		
26 days vs. 8.0 kg (*n* = 112)	FLF ^b^ (*Lactococcus lactis* subsp. *cremoris* 303)		↓ carcass weight, ↓ DMI vs. dry pelleted feed (d 0 to slaughter)		
22.56 ± 2.55 days (*n* = 48)	Fresh LF	2.5:1	↑ ADFI, ↑ ADG, ↓ FCR vs. dry pellets	Pigs were fed the treatments for 28 days in the two feeding trials	[50]
22.56 ± 2.55 days (*n* = 98)	Fresh LF		↑ ADFI, ↑ ADG, ↓ FCR vs. dry pellets		
28 days vs. 7.9 ± 1.0 kg (*n* = 30)	FLF (Bactocell^®^ added to water and feed)	2.5:1	↓ BWG during week 4 of the trial vs. dry feed	FLF was produced with Bactocell^®^ with 50% daily backslopping. The experimental period was 4 weeks. The diet in the second trial contained 10 g/kg sepiolite to improve homogeneity of the liquid feed and avoid sedimentation	[65]
27 days vs. 6.3 ± 1.2 kg (*n* = 45)	FLF (Bactocell^®^ already added to dry diet before mixing)		↑ DM intake, ↑ BWG, ↑ F:G vs. dry feed and dry feed with Bactocell^®^		
28 ± 1 days vs. 8 ± 1.1 kg (*n* = 120)	FLF	2.5:1	↓ ADG, ↓ ADFI, ↓ G:F vs. dry meal (d 1 to 42)	FLF and FLC were fermented at 20°C with 50% backslopping 3 times daily. The experimental period began after 5 days of fermentation and lasted for a period of 6 weeks	[43]
	FLC ^c^		↓ ADG, ↓ ADFI, ↓ G:F vs. dry meal (d 1 to 42)		
28 days vs. 7.9 ± 1.1 (*n* = 20)	Fresh LF	2.75:1	Numerical ↑ ADG and FCR vs. FLF (NSD)	FLF was fermented for 8 h with 50% backslopping. The experimental period lasted for a period of 4 weeks	[34]
21.0 ± 2.8 days vs. 5.7 ± 0.7 kg (*n* = 72)	Fresh LF-10 d	3: 1	↑ ADG, ↑ ADFI vs. dry feed (up to d 40)	Fresh LF-10 d was fed for 10 days followed by dry feed for 30 days vs. control (dry feed for 40 days)	[94]
	Fresh LF-20 d		↑ ADFI vs. dry feed, ↓ FCE vs. dry feed and fresh LF-10 d (up to d 40)	Fresh LF-20 d was fed for 20 days followed by dry feed for 20 days vs. control (dry feed for 40 days)	
28 days vs. 7.3 ± 0.3 kg (*n* = 136)	FLF	2.5:1	↑ BW, ↑ ADG, ↑ ADFI, ↓ FCR vs. dry feeding	FLF was mixed and then soaked for 15 h before feeding. No backslopping was performed with a new batch prepared for feeding the next day. The experimental period was 26 days	[51]
24 ± 4 days vs. 7 ± 1 kg (*n* = 48)	FLF (Bactocell^®^)	2.5:1	NSD in ADFI, ADG, FCR vs. control (on d 28) vs. control (but numerically improved for FLF)	Control diet was lactic acid-supplemented FLF. The experimental period lasted 28 days	[69]
28 days vs. 7.4 ± 0.4 kg (*n* = 72)	SLF ^d^	2.5:1	↑ ADFI, ↑ ADG, ↑ FCR vs. dry feed	Feed was soaked for 1 h before feeding. Soaking for 24 h did not improve performance and supplementation with 300 ppm xylanase reduced feed intake. The experimental period lasted 26 days	[102]
**Grow-finisher pigs**					
31 ± 3.5 kg (*n* = 60)	Fresh LF	2.5:1	↑ ADG vs. FLF, ↑ ADFI vs. FLF and dry feed	FLF was fermented at 20°C for 4 days before the trial with 50% backslopping at each feeding. G:F was similar for all diets. Performance parameters were recorded fortnightly up to final BW of 101 ± 4.0 kg	[11]
	FLF		↓ ADG vs. fresh LF and dry feed, ↓ ADFI vs. fresh LF		
49.2 ± 0.68 kg (*n* = 64)	Fresh LF	3:1	↑ Final LW, ↑ ADG, ↑ Lean tissue growth rate vs. dry pellets	Trial period was 6 weeks of unrestricted feeding	[4]
47.1 ± 1.55 kg (*n* = 64)	Fresh LF	3:1	↑ Final LW, ↑ ADG, ↑ ADFI, ↑ FCR vs. dry pellets	Trial period was 6 weeks of restricted feeding (5% to 10% below *ad libitum* intake).	
16 weeks vs. 54.3 ± 6.3 kg (*n* = 32)	Fresh LF	2.5:1	NSD between treatments for ADG or FCR	FLF was inoculated with *Lactobacillus salivarius*, Bactocell^®^ or Stabisil^™^ and fermented for 24 h at 30 °C	[99]
	FLF (*Lactobacillus salivarius*)				
	FLF (Bactocell^®^)				
	FLF (Stabisil^™^)				
9-11 weeks vs. 18.3–29.6 kg (*n* = 122)	Fresh LF	1.5, 2.5, 4:1	↑ ADG, ↑ FCR vs. dry diet	Treatments of fresh liquid feed with water:feed ratios of 1.5, 2.5, 4:1 were compared to dry feeding, where 2.5:1 resulted in better ADG and FCR	[103]
32.7 kg (*n* = 432)	Fresh LF (meal)	2.5:1	↑ BW, ↑ ADG, ↓ G:F vs. dry and wet/dry meal	Two experiments were carried out with batches of 216 pigs. Both experimental periods lasted 64 days from 32.7 to 100 kg	[39]
	Fresh LF (pellets)		↑ BW, ↑ ADG vs. dry pellets, ↓ G:F vs. dry and wet/dry pellets		
29.8 ± 0.92 (*n* = 216)	Fresh LF	2.5:1	↑ ADG vs. FLF, similar FCE to wet/dry (lowest)	The experimental period lasted 68 days prior to slaughter (29.8 ± 0.92 kg to 102.3 ± 0.76 kg). For FLF the whole diet was fermented prior to feeding, while for FLC only the cereal component of the diet was fermented and then mixed with the balancer and water prior to feeding.	[68]
	FLF (Sweetsile^®^)		↓ BW, ↓ ADG, ↓ FCE vs. fresh LF, FLC and wet/dry, ↑ ADFI vs. wet/dry		
	FLC (Sweetsile^®^)		↑ ADFI vs. wet/dry, ↑ ADG, ↑ FCE vs. FLF		
85.3 ± 1.69 kg (*n* = 160)	Fresh LF	2.5:1	↑ BW, ↑ ADG vs. wet/dry	The experimental period lasted 26 days prior to slaughter (85.3 ± 1.69 kg to 117.5 ± 0.72 kg). Diets were prepared as above	
	FLF (Sweetsile^®^)		↓ BW, ↓ ADG vs. FLC, ↓ ADFI vs. FLC and wet/dry, ↓ FCR vs. wet/dry		
	FLC (Sweetsile^®^)		↑ BW, ↑ ADFI vs. FLF and wet/dry, ↑ ADG vs. fresh LF, FLC and wet/dry		
33.4 ± 0.88kg (*n* = 392)	Fresh LF (ENZ ^e^ −)	2.5:1	Interaction between ENZ and soaking for ADG (d 21), ↑ LW (0.8 kg) for SLC vs. fresh LF (d 21), ↑ fat depth and lower lean meat % for ENZ+ vs. ENZ (d 70), ↑ ADG for SLC (ENZ+) vs. fresh LF (ENZ+) at d 21	For fresh LF, the whole diet (including ENZ for ENZ+) was mixed with water and fed after 5 min mixing. For SLC diets, the cereal component of the diet (including ENZ for ENZ+) was mixed with water and agitated for 3 h, mixed with the balancer fraction for 5 min and fed out. The experimental period lasted 71 days	[32]
	Fresh LF (ENZ+)				
	SLC ^f^ (ENZ−)				
	SLC (ENZ+)				
40.6 ± 0.50 kg (*n* = 252)	Fresh LF (ENZ−)	2.5:1	Interaction between FLC and ENZ for LW (d 28 and d 55), ↑ LW for FLC (ENZ−), ↑ ADG for FLC (4.1%) vs. fresh LF, ↑ FCR for ENZ+ diets (3.8% lower), ↑ carcass weight (2.0 kg) and ↓ (1.0%) lean meat % for FLC diets (d 55)	The experimental period lasted 55 days. Fresh LF diets were prepared as above. For FLC diets the cereal component of the diet (including ENZ for ENZ+) was mixed with water, inoculated with Sweetsile^®^, and fermented for 52 h, with daily backslopping. The balancer fraction was added to the fermented cereal and mixed for 5 min before feed out	[33]
	Fresh LF (ENZ+)				
	FLC (Sweetsile^®^, ENZ−)				
	FLC (Sweetsile^®^, ENZ+)				
61 ± 2 days vs. 20.8 ± 2.06 kg (*n* = 20)	FLF (*Lactobacillus plantarum*, *Pediococcus pentosaceus* and *Lactococcus lactis*) vs. fresh LF	NR ^h^	Numerical ↑ ADFI in FLF (NSD), Numerical ↓ FCR in FLF (NSD)	Fresh LF was mixed with water immediately before feeding, while for FLF the whole diet was fermented with *Lactobacillus plantarum*, *Pediococcus pentosaceus* and *Lactococcus lactis* for 24 h. The experimental period lasted 28 days	[100]
	PFLF ^g^ (with non-fermented course cereals) vs. fresh LF			For PFLF, rapeseed extracted meal and part of the rye (60% of the whole diet) were fermented as above while the remaining cereal components were not fermented. Phytases and the mineral supplement were added after fermentation. Fresh LF was prepared as above but with phytases added. The experimental period lasted 28 days	

↑ denotes an improvement in a parameter compared to the specified treatment group; ↓ denotes poorer performance in the parameter compared to the specified treatment group. ^a^ Fresh LF = Fresh liquid feed: all dietary ingredients are mixed with water and fed out immediately. ^b^ FLF = Fermented liquid feed: all dietary ingredients are fermented for a specific period of time prior to feeding. ^c^ FLC = Fermented liquid cereal: only the cereal fraction of the diet is fermented, with the remaining dietary ingredients added prior to feeding. ^d^ SLF = Soaked liquid feed: The whole diet was soaked for 1 h before feeding. ^e^ ENZ = Xylanase and β-glucanase enzyme complex (Rovabio Excel AP, Adisseo France SAS, Antony, France); +/− indicates whether diets were supplemented (+) or not (−) with ENZ. ^f^ SLC = Soaked liquid cereal: cereal fraction of the diet soaked in water for 3 h prior to mixing with balancer fraction (soybean meal, synthetic amino acids, minerals and vitamins) immediately prior to feeding. ^g^ PFLF = Partially fermented liquid feed: rapeseed extracted meal and part of the rye (60% of the whole diet) were fermented while the remaining cereal components were not fermented. ^h^ NR: Not reported. ^i^ Performance parameters: BW: Body weight; ADFI: Average daily feed intake; ADG: Average daily gain; G:F: Gain to feed ratio; FCE: Feed conversion efficiency; DMI: Dry matter intake; LW: live weight; Lean meat %: Lean meat percentage; NSD: No statistical difference; ALF: Acidified liquid feed; FCR: Feed conversion ratio; BWG: Body weight gain; F:G: Feed to gain ratio.

## Data Availability

Not applicable.
